# Perceived Behavioural Control and Animal-Welfare Ethics Predict Cultured-Meat Acceptance in INDIA: An Extended Theory of Planned Behaviour Analysis

**DOI:** 10.3390/foods15142474

**Published:** 2026-07-13

**Authors:** Nematullah Farooqui, Anna M. Kaczmarek

**Affiliations:** Department of Food Quality and Safety Management, Faculty of Food Science and Nutrition, Poznań University of Life Sciences, Wojska Polskiego 31, 60-624 Poznań, Poland; nematullahfarooqui84@gmail.com

**Keywords:** cultured meat, consumer acceptance, theory of planned behaviour, India, structural equation modelling, alternative proteins

## Abstract

Cultured meat is proposed as a sustainable protein alternative, yet consumer acceptance remains one of the principal barriers to its commercialisation, and the evidence base remains overwhelmingly Western. India—the world’s most populous nation, characterised by dietary pluralism and the principle of ahimsa—remains understudied. Using a cross-sectional online survey, this study applied an extended Theory of Planned Behaviour to identify psychological predictors of the intention to try cultured meat among English-speaking Indian consumers (*N* = 255), employing latent-variable structural equation modelling with a robustness check for common-method variance. Perceived behavioural control was the strongest predictor of intention (β = 0.609, *p* < 0.001), followed by attitude (β = 0.295) and subjective norms (β = 0.263, sensitive to method variance); however, dominance decomposition attributed the largest share of explained variance to subjective norms. Ethical concern for animal welfare was the strongest predictor of attitude (β = 0.487), while disgust was a significant negative predictor (β = −0.272); perceived unnaturalness, health concern, and dietary-group differences were non-significant. The model explained 69.9% of the variance in intention. Acceptance reflected perceived feasibility and ethical concern for animal welfare rather than perceived unnaturalness, diverging from Western evidence and carrying implications for marketing and certification strategies.

## 1. Introduction

Providing adequate protein for a global population projected to reach approximately 10 billion by 2050 is one of the central challenges of contemporary food systems [1]. Conventional meat production is a major source of high-quality dietary protein, but it is also associated with considerable environmental costs, including high land and water use, greenhouse-gas emissions, and zoonotic risks; these pressures are intensifying as demand for animal protein continues to rise [2]. Cultured meat, produced from animal cells cultivated in bioreactors without the slaughter of live animals, has been advanced as one possible response. It is biologically similar to conventional meat but is generated through a controlled biomanufacturing process. Despite substantial technological progress, the primary impediment to cultured-meat commercialisation has shifted from biological feasibility to behavioural acceptance, namely, consumer willingness to accept the product [2,3].

Research on the psychological factors influencing cultured-meat acceptance has expanded substantially over the past decade, identifying a limited number of recurrent predictors. Recent reviews have synthesised this rapidly growing literature, underscoring the roles of awareness, information provision, and familiarity in shaping acceptance [4,5], while early analyses of Western media coverage noted a disproportionate focus on vegetarian reactions, with implications for cultured-meat marketing [6]. The Theory of Planned Behaviour (TPB) [7] has been the dominant framework, positing that behavioural intention is jointly shaped by attitude toward the behaviour, subjective norms, and perceived behavioural control. Extended TPB models tailored to cultured meat further include perceived unnaturalness and disgust as cognitive and affective barriers [8,9], ethical concern for animal welfare as a motivational antecedent [2,10], and health-related concerns as a potentially ambivalent predictor. Recent studies applying the extended TPB to cultured meat in Western settings have generally reported attitude as the strongest predictor of intention, with perceived behavioural control playing a secondary role [11,12]. However, the extant evidence base is drawn overwhelmingly from Europe and North America, with comparatively little empirical work on Asian—and particularly Indian—consumers. Recent studies have extended this evidence base to further contexts, including China [13], Japan and China [14], Japan [15], Greece [16], Arab countries [17], and a cross-cultural comparison of Belgium, Chile, and China [18]. Engel et al. [19] subsequently extended the cultured-protein acceptance literature to a large Nordic sample (Denmark, Finland, and Norway; *N* = 3862), confirming the role of beliefs about industry necessity, food innovativeness, and social-network attitudes in shaping behavioural intentions toward cultured meat, fish, and dairy, while Maqsood et al. [20] applied an attitudes–norms–control framework to a representative sample of 1666 Emirati nationals and reported that subjective norms and perceived behavioural control jointly shape willingness to substitute conventional animal protein with alternatives including cultured meat. India, however, remains conspicuously underrepresented in this rapidly expanding evidence base.

India occupies a distinctive position in the global protein-transition debate. As the world’s most populous nation, with a rapidly expanding middle class and increasing demand for animal-derived protein, the trajectory of meat consumption in India will have considerable global consequences. At the same time, the Indian food system is shaped by deeply entrenched cultural and religious frameworks that lack direct parallels in most Western contexts. The principle of ahimsa—from Sanskrit a- (“non-”) and hiṃsā (“violence”)—is a foundational ethical principle in Hindu, Jain, and Buddhist traditions that enjoins non-violence toward all sentient beings; in dietary practice, it underpins vegetarianism as an expression of moral compassion and supports one of the largest vegetarian populations worldwide. Cultured meat is positioned ambiguously relative to this framework: although it is biologically meat, it does not require the slaughter of a live animal, which allows it to be interpreted either as a transgression of the vegetarian/non-vegetarian distinction, because it remains biologically animal-derived, or as a technological extension of ahimsa-aligned compassion, because it eliminates the suffering inherent in conventional meat production. Muslim dietary practices, including halal requirements, together with national food-labelling regulations for vegetarian foods, further structure what may be classified as acceptable food. These cultural particularities suggest that the psychological predictors identified in Western research may operate differently in the Indian context, both in their relative importance and in their meaning. However, empirical evidence remains limited. Cross-country studies have incorporated relatively small Indian subsamples [21], and dedicated Indian research has tended to focus on specific regions or informed consumer segments. A more recent contribution from this research line by Choudhary et al. [22] surveyed 400 food science scholars and veterinary professionals in Jammu and Kashmir and reported substantially higher perceptions of unnaturalness (86%) and lower endorsement of the animal-welfare argument (42%) than have typically been observed in Western general-consumer samples, motivating the present theory-driven analysis of how such perceptions translate into behavioural intention in an English-speaking general-adopter sample. A recent comparative study of consumer trust in emerging food technologies across Croatia and India [23] likewise demonstrated that trust in technologies such as lab-grown meat is strongly culturally contingent, yet it did not model the psychological pathway from such perceptions to behavioural intention within a theory-driven framework. Consequently, a theory-driven empirical analysis of the psychological predictors of cultured-meat acceptance among English-speaking Indian consumers is timely and necessary.

**Figure 1:**
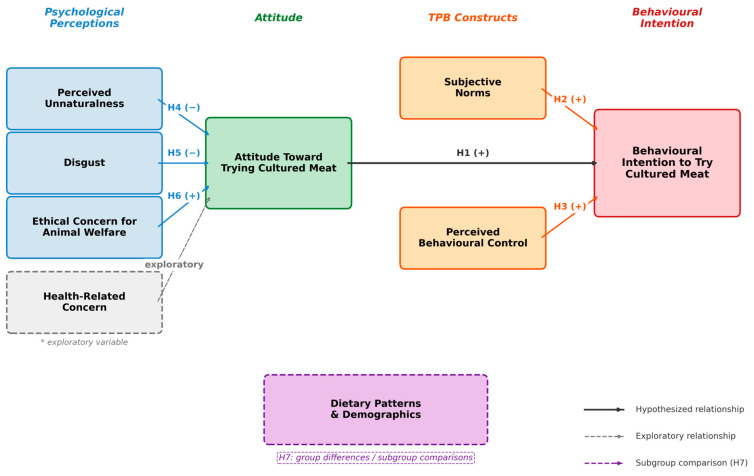
Conceptual research model (extended Theory of Planned Behaviour), * refers to exploratory variable.

The present study addresses this gap by applying an extended TPB framework to a sample of English-speaking Indian adult consumers (*N* = 255). The restriction to English-speaking respondents reflects both practical and substantive considerations: a singles English-language instrument permits standardised measurement across a linguistically heterogeneous national population, while English-speaking Indian consumers constitute the demographic most likely to encounter cultured meat upon market entry, given their concentration in urban centres, their higher average educational and socioeconomic profile, and their digital connectivity, which together qualify them as a likely early-adopter segment for this novel food technology. The generalisability implications of this restriction are addressed in Section 4.6. The model integrated the three core TPB predictors of behavioural intention—attitude, subjective norms, and perceived behavioural control—with four extended psychological antecedents of attitude: perceived unnaturalness, disgust, ethical concern for animal welfare, and health-related concern. The study tested seven hypotheses (H1–H7) and one research question (RQ1): H1–H3 examined the core TPB predictors of intention; H4–H6 examined perceived unnaturalness (predicted negative), disgust (predicted negative), and ethical concern (predicted positive) as predictors of attitude; H7 examined whether attitudes and intentions differ across dietary patterns; and RQ1 asked whether health-related concern negatively influences attitude. The hypothesised paths and the research question (Figure 1) were tested using latent-variable structural equation modelling, complemented by a pre-specified package of robustness and triangulation analyses (Section 2.5). Beyond extending the cultured-meat acceptance literature to a substantially understudied population, the present analysis contributes two structural tests that this literature has not previously addressed in an Indian context: whether the relative weight of attitude, subjective norms, and perceived behavioural control in shaping intention departs from the attitude-dominant pattern reported in Western samples, and whether attitudes and intentions vary systematically across dietary identity groups (vegetarian, flexitarian, omnivore, vegan) in a population where dietary identity is institutionally codified. The role of dietary identity in cultured-meat acceptance has recently received direct experimental investigation in Western samples (Lewisch and Riefler [24]); the present study extends this line of investigation to an Indian context in which dietary identity carries additional moral and religious significance. The findings are positioned against the Western evidence base, with implications for marketing, certification, and consumer-education strategies in the Indian market.

## 2. Materials and Methods

### 2.1. Study Design

The present study employed a quantitative, cross-sectional research design Data were collected through a self-administered online questionnaire hosted on Google Forms (Google LLC, Mountain View, CA, USA). The instrument was developed on the basis of an extended Theory of Planned Behaviour framework adapted to cultured-meat acceptance and was refined iteratively through a pilot phase before deployment in the main study.

### 2.2. Pilot Study

A pilot study (*N* = 64) was conducted before the main data collection to evaluate the clarity, comprehensibility, internal flow, and completion time of the instrument. The pilot participants were drawn primarily from the researcher’s extended personal and professional networks, and the pilot instrument was administered bilingually in English and Hindi. The pilot phase indicated that the original questionnaire was excessively long, which was associated with respondent fatigue. In response, the instrument was substantially shortened. The four-item Attitude scale was retained in full; the originally longer Subjective Norms, Perceived Behavioural Control, and Intention scales were each reduced to the two best-performing items, selected on the basis of pilot item-total correlations and face-validity ratings. The four single-item antecedents of attitude (perceived unnaturalness, disgust, ethical concern for animal welfare, and health-related concern) were retained as in the pilot version. Minor refinements were also made to item wording to enhance clarity, and the introductory description of cultured meat in Section B was rewritten to remove valence-laden phrasing identified by pilot respondents.

### 2.3. Participants and Procedure

The target population comprised adult residents of India with sufficient English proficiency to complete an online questionnaire. A non-probability convenience sampling strategy, supplemented by elements of snowball sampling, was adopted. The questionnaire was distributed electronically in December 2025 through social media platforms—including Instagram, WhatsApp, LinkedIn, and Facebook—in addition to personalised email invitations. Recipients were invited to forward the questionnaire to other potential respondents. No restrictions were imposed on age, gender, dietary pattern, or geographic location within India.

Before beginning the questionnaire, respondents were informed of the purpose of the study, the voluntary and anonymous nature of participation, and their right to withdraw at any time without consequence. Proceeding to the first question constituted informed consent. The study was conducted in accordance with the ethical principles of the Declaration of Helsinki [25] and with the institutional ethical guidelines of the Poznań University of Life Sciences. Under Polish national legislation and the relevant University guidelines, formal ethics-committee approval was not required for this anonymous, non-interventional online questionnaire study, in which no identifiable personal data, sensitive personal data, biological samples, food tasting, supplementation, medical intervention, clinical procedure, or experimental manipulation were collected or performed; the full Institutional Review Board Statement, including the relevant statutory citations, is provided in the back matter.

A total of 259 responses were obtained. After listwise deletion to address missing values on the key analytic variables, the final analytic sample comprised *N* = 255 complete cases. Missingness was minimal and non-systematic (<1% per variable); missing values occurred exclusively on the four single-item perceptual measures (perceived unnaturalness, disgust, ethical concern, and health-related concern), and each of the four excluded cases omitted at least one of these items. The achieved sample size satisfied both the absolute minimum of 200 observations and the 10:1 ratio criterion conventionally recommended for structural equation modelling with maximum likelihood estimation [26]. Beyond these conventional rules of thumb, statistical power to detect each individual structural path was evaluated through a model-based post hoc sensitivity power analysis, following Cohen [27] and tailored to the observed effect sizes and model degrees of freedom (Section 2.5). This analysis reports the minimum detectable effect at conventional power thresholds for every hypothesised structural relationship and provides the basis for the inconclusive-versus-substantive-null distinction discussed in Section 3.5.

### 2.4. Measures

The final instrument comprised eight sections. Section A consisted of a single screening item on prior awareness of cultured meat (binary: Yes/No). Section B presented a neutrally worded informational note describing the production process and characteristics of cultured meat, in line with the informed-choice paradigm widely adopted in this literature [3]; the framing of such information has itself been shown to affect cultured-meat acceptance [28]. Sections C through F contained the core TPB constructs. Attitude toward trying cultured meat was measured by four semantic differential items rated on 7-point scales (negative–positive, unpleasant–pleasant, unattractive–attractive, and not worthwhile–worthwhile), following Ajzen [29]; all four items were oriented so that higher scores denoted a more positive attitude, and no reverse-coding was required. Subjective norms were measured by two 7-point Likert items (1 = strongly disagree, 7 = strongly agree) capturing the injunctive component of perceived social approval. Perceived behavioural control was measured by two 7-point Likert items assessing perceived ease of access and personal autonomy, in line with Ajzen’s [30] treatment of perceived behavioural control and self-efficacy. Behavioural intention was measured by two 7-point Likert items addressing general intention and intention to purchase, contingent on availability and reasonable price.

Section G assessed four psychological antecedents of attitude through single-item 7-point Likert measures: perceived unnaturalness, disgust, ethical concern for animal welfare, and health-related concern. The use of single-item measures for these four constructs is consistent with established practice in cultured-meat acceptance research [8,9,10] and with the standard psychometric criteria for justified single-item measurement, namely: (i) double concreteness, in that both the object (cultured meat) and the attribute (e.g., “unnatural” or “disgusting”) are concrete and unambiguous; (ii) singularity rather than multidimensionality, so that a respondent’s evaluation can be adequately captured by a single direct question; and (iii) bounded scope, so that no summative averaging across qualitatively distinct facets is required. Under these conditions, multi-item scales offer little incremental construct validity over a single well-formulated item while increasing respondent burden, which the pilot phase (Section 2.2) identified as the principal threat to data quality. The single-item operationalisation of these particular antecedents is also the predominant choice in the cultured-meat acceptance literature, supporting cross-study comparability. Section H collected demographic and background information, including age, gender, dietary pattern (omnivore, flexitarian, vegetarian, and vegan), country of residence, education level, place of residence, and self-rated financial situation. The dietary-pattern item used the general label “vegetarian” without distinguishing lacto- from lacto-ovo-vegetarian diets; this lack of granularity, which has particular cultural significance in the Indian context, is acknowledged as a limitation.

The items were adapted from established TPB instruments for cultured-meat and novel-food acceptance research [3,7,8,9]. The full instrument is available in the Supplementary Materials.

### 2.5. Statistical Analysis

All statistical analyses were conducted in Python 3.13, using the following libraries: numpy 2.4.2, scipy 1.17.0, pandas 3.0.0, statsmodels 0.14.6, scikit-learn 1.8.0, pingouin 0.5.5, semopy 2.3.11 (for structural equation modelling), and PyMC 6.0.1 with pytensor 3.0.3 and arviz 1.1.0 (for Bayesian path modelling). Figures were generated with matplotlib 3.10.8 and seaborn 0.13.2. The significance threshold was set at α = 0.05 for all inferential tests.

Composite scores for the four multi-item TPB constructs were computed as the arithmetic mean of the constituent items. Internal consistency was assessed by Cronbach’s α and, given the documented limitations of α under unequal factor loadings, by McDonald’s ω estimated from confirmatory factor analysis loadings [31]. Descriptive statistics were computed for all study variables, and distributional properties were evaluated using the Shapiro–Wilk test. Pearson and Spearman correlations were computed in parallel to assess the robustness of bivariate associations under potential non-normality and the ordinal scaling of Likert-type items. Variance inflation factors were inspected to evaluate multicollinearity, with the conventional threshold of VIF < 5 adopted [32].

The hypothesised model was tested using latent-variable structural equation modelling with maximum likelihood estimation. The measurement model specified four latent constructs (Attitude, Subjective Norms, Perceived Behavioural Control, and Intention), each indicated by its respective items, while the four psychological antecedents of attitude were retained as observed exogenous predictors. Global model fit was assessed using the χ^2^ statistic, the Comparative Fit Index (CFI), the Tucker-Lewis Index (TLI), the Root Mean Square Error of Approximation (RMSEA), and the Standardised Root Mean Square Residual (SRMR), with the conventional thresholds recommended by Bentler and Bonett [33], Hu and Bentler [34], and Kline [26] adopted.

In view of the single-source self-report design, common-method variance was assessed as a potential threat to construct validity [35]. The Unmeasured Latent Method Construct (ULMC) approach was employed as a post hoc robustness check [36]. A latent method factor was added to the baseline model and constrained to load on all multi-item indicators while remaining orthogonal to the substantive constructs. Indirect effects of the four perceptual predictors on intention through attitude were estimated using the percentile bootstrap method with 5000 resamples; an indirect effect was deemed significant when its 95% confidence interval excluded zero [37,38].

Group differences in attitude and intention across dietary patterns (H7) were tested using a multi-method approach. A multivariate analysis of variance (MANOVA) with Pillai’s Trace was used to test the joint vector of attitude and intention, selected for its robustness to violations of multivariate normality and unequal group sizes [39]. One-way analysis of variance with Tukey HSD post hoc comparisons was conducted separately for attitude and intention, with effect sizes reported as η^2^, ω^2^, and Cohen’s f [27]. Non-parametric Kruskal–Wallis tests, including all four dietary groups, served as sensitivity checks. The Vegan subgroup (*n* = 4) was excluded from the parametric tests owing to insufficient size but was retained in the non-parametric analyses.

Robustness and Triangulation Analysis. The primary confirmatory analysis was the latent structural equation model with Holm–Bonferroni multiplicity correction and the ULMC robustness check (Section 2.5). To evaluate the robustness of these primary results and triangulate the findings across statistical paradigms, a pre-specified set of secondary analyses was undertaken. These secondary analyses were planned before the estimation of the main structural model but were not formally preregistered. All analyses were conducted on the same analytic sample (*N* = 255) and were implemented in Python (version 3.13). The complete reproducibility package, including data, scripts, and a pinned requirements file, is available in the Supplementary Materials.

Confirmatory versus exploratory tests were pre-classified. Hypotheses H1 to H6 and H7 were treated as confirmatory; RQ1 (health-related concern) was analysed separately and is reported with this label throughout.

Item-level psychometric diagnostics. For each multi-item scale, Cronbach’s α-if-item-deleted and the corrected item-total correlations were computed in addition to the scale-level α and McDonald’s ω reported above.

Multiplicity correction. Within the confirmatory family H1–H6, raw *p*-values from the latent structural paths were adjusted using the Holm–Bonferroni procedure at a familywise α of 0.05. The Benjamini–Hochberg false discovery rate procedure (q = 0.05) was applied to RQ1.

Bootstrap robust standard errors. The latent structural model was re-estimated in 1000 non-parametric bootstrap resamples (resampled with replacement; case-level resampling). For each structural path, the bootstrap mean, bootstrap standard error, percentile and bias-corrected 95% confidence intervals, and a two-sided bootstrap *p*-value were computed. Replicates yielding standardised coefficients with absolute value above 1.5 were treated as numerical failures (arising from near-singular factor loadings in the resampled covariance structure) and were excluded from the summary. The number of stable replicates is reported for every path.

Sensitivity power analysis. For every structural path, a post hoc Wald-type sensitivity power analysis was conducted using the bootstrap-derived standard error as a realistic estimate of the path uncertainty. Achieved power was computed at the uncorrected α of 0.05 and at the most conservative Holm-corrected α of 0.05/6. The minimum detectable standardised effect at power = 0.80 was reported for each path under both α thresholds. This analysis was used to distinguish substantive null findings from findings whose non-significance may reflect inadequate power.

Binary-outcome sensitivity. The continuous intention measure was dichotomised at the scale midpoint (intention > 4 indicating willingness to try cultured meat). A logistic regression with heteroskedasticity-consistent (HC3) robust standard errors was estimated to test the core TPB predictors against the dichotomised outcome. The same procedure was applied to a dichotomised attitude outcome for the H4–H6/RQ1 sensitivity model. Odds ratios with 95% confidence intervals, McFadden’s pseudo-*R*^2^, and the area under the receiver operating characteristic curve (*AUC*) are reported.

Dominance analysis. The relative importance of the predictors in two ordinary least-squares models—intention regressed on the three core TPB constructs, and attitude regressed on the four perceptual predictors—was quantified by Budescu’s general dominance decomposition [40]. For every predictor the average incremental *R*^2^ across all 2^p−1^ subsets of co-predictors was computed and normalised to the full-model *R*^2^. This procedure provides an order-independent decomposition of explained variance and is reported alongside the structural-model coefficients to confirm the relative-importance ranking.

Triangulation across estimation paradigms. Three independent paradigms were used to confirm the patterns identified by covariance-based SEM. First, a Bayesian path model with weakly informative priors (b~Normal(0, 1) on standardised coefficients) was estimated in PyMC with four chains of 2000 tuning and 2000 sampling iterations. Posterior 95% credible intervals, posterior probability of direction, and Savage–Dickey approximations of the Bayes factor *BF*_10_ against a point-null at zero are reported. Second, an elastic net regression (l1-ratio and penalty tuned by 10-fold cross-validation) and a random forest regressor with permutation importance were estimated for both outcomes (intention and attitude). Predictors retained by the elastic net and ranked highly by random forest permutation importance were compared with the set of predictors identified by the structural model. Third, the same path model was re-estimated under the variance-based partial least squares structural equation modelling (PLS-SEM) paradigm using a transparent in-house implementation of the canonical Wold–Tenenhaus algorithm with Mode A outer estimation and the path-weighting inner scheme. Five hundred non-parametric bootstrap resamples were used for inference on the PLS path coefficients. Convergence of the path estimates across the covariance-based SEM, the Bayesian path model, the regularised and non-parametric machine learning analyses, and the PLS-SEM analysis was treated as triangulation evidence for the structural conclusions.

The triangulation strategy adopted in this study, which combines covariance-based latent SEM with Bayesian path modelling, partial least squares SEM, and regularised machine learning estimation, was motivated by two epistemic considerations. First, the present analysis was designed to distinguish substantively supported null findings from inconclusive non-significance—a distinction that frequentist significance testing cannot make on its own and that requires Bayesian evidence ratios alongside post hoc power. Second, partial least squares and regularised machine learning estimation provide estimation paradigms with mathematical assumptions that differ from those of maximum likelihood SEM; the convergence (or divergence) of structural conclusions across these paradigms constitutes a methodological safeguard against the conclusions being an artefact of any single estimator. The four paradigms therefore play complementary epistemic roles rather than constituting redundant repetitions of the same analysis. Two further sensitivity analyses were conducted as additional robustness checks on the Perceived Behavioural Control findings. To assess the sensitivity of the Perceived Behavioural Control effect to its two-item parameterisation, the latent structural model was re-estimated twice, with Perceived Behavioural Control represented by each of its two indicators alone (single-indicator specifications, with the item treated as a perfect measure of the construct). To address the high collinearity between Subjective Norms and Perceived Behavioural Control, an alternative hierarchical model specifying Subjective Norms as an antecedent of Perceived Behavioural Control (Subjective Norms → Perceived Behavioural Control → Intention) was estimated and compared against the parallel baseline model using the Akaike and Bayesian information criteria.

Generative AI statement. The authors used a generative AI assistant (Claude Opus 4.7, Anthropic PBC, San Francisco, CA, USA) to assist with the restructuring and formatting of the Python analysis code and with the code used to generate the manuscript figures. The study design, data collection, statistical analyses, results, and their interpretation were carried out by the authors; all AI-assisted code was independently executed and verified against the reported outputs, and the authors take full responsibility for the content.

## 3. Results

### 3.1. Sample Characteristics

Of the 259 questionnaires submitted, 4 cases with missing values on the key analytic variables were excluded by listwise deletion, yielding a final analytic sample of *N* = 255 complete cases (<1% missingness per variable). The demographic profile of the sample is presented in Table 1. Respondents were predominantly male (*n* = 177; 69.4%), with the remainder identifying as female (*n* = 73; 28.6%) or preferring not to disclose their gender (*n* = 5; 2.0%). The educational profile of the sample was substantially skewed toward tertiary attainment: 96.1% of respondents held a bachelor’s degree or higher, with 56.1% holding a bachelor’s (*n* = 143) and 40.0% a master’s degree or above (*n* = 102). Urban residence dominated the place-of-residence distribution, with 80.4% residing in cities of at least 100,000 inhabitants and 42.4% (*n* = 108) in cities of more than 500,000. All respondents reported India as their country of residence.

The dietary distribution comprised 63.1% omnivores (*n* = 161), 28.6% flexitarians (*n* = 73), 6.7% vegetarians (*n* = 17), and 1.6% vegans (*n* = 4). The very small size of the vegan subgroup precluded its inclusion in the parametric tests for Hypothesis 7 but was retained in the non-parametric sensitivity analyses. Prior awareness of cultured meat was reported by 45.1% of respondents (*n* = 115), with 54.9% (*n* = 140) indicating that they had not previously encountered the concept before participating in the survey.

The demographic profile of the present sample diverges substantially from that of the broader Indian adult population. Indian adults are approximately half male and the great majority do not hold a tertiary qualification, whereas the present sample is 69.4% male and 96.1% tertiary-educated. Approximately one-third of the Indian population resides in urban areas, whereas 80.4% of the present respondents reside in cities of at least 100,000 inhabitants. Additionally, the sample was restricted to respondents with sufficient English proficiency to complete the questionnaire, which over-represents English-literate urban adults. The achieved sample is therefore best characterised as an urban, tertiary-educated, English-literate, predominantly male early-adopter segment of the Indian consumer market rather than as a representative cross-section of Indian adult consumers; all subsequent results are interpreted accordingly.

### 3.2. Measurement Reliability and Descriptive Statistics

The internal consistency of the multi-item scales is reported in Table 2. The Attitude scale demonstrated excellent reliability (Cronbach’s α = 0.949; McDonald’s ω = 0.950), with item-total correlations exceeding 0.85 for all four indicators and α-if-item-deleted values that did not rise above the full-scale α for any indicator (range: 0.929–0.941; full item-level diagnostics in Table S1), confirming that no item warranted exclusion. The Subjective Norms scale (α = 0.934; ω = 0.936; inter-item *r* = 0.876) and the Intention scale (α = 0.908; ω = 0.909; inter-item *r* = 0.831) both exhibited strong internal consistency. The Perceived Behavioural Control scale yielded acceptable reliability for a two-item measure (α = 0.698; ω = 0.699; inter-item *r* = 0.536), essentially at the conventional 0.70 threshold for established scales and with an inter-item correlation comfortably above the 0.50 threshold. The four perceptual constructs (G1–G4) were measured by single items and are therefore not eligible for internal-consistency estimation.

Descriptive statistics for all study variables are reported in Table 2. Attitude (*M* = 3.36, *SD* = 1.84) and Subjective Norms (*M* = 3.47, *SD* = 2.01) were positioned below the scale midpoint, indicating that initial evaluations of cultured meat were, on average, modestly unfavourable. Intention to try cultured meat was similarly below the midpoint (*M* = 3.57, *SD* = 2.10). In contrast, Perceived Behavioural Control was positioned slightly above the midpoint (*M* = 4.20, *SD* = 1.93), suggesting moderate perceived feasibility of trying cultured meat. Among the perceptual predictors, Health Concern recorded the highest mean (*M* = 5.39, *SD* = 1.91), with Unnaturalness (*M* = 4.59, *SD* = 2.16) and Ethics (*M* = 4.57, *SD* = 2.08) positioned above the midpoint and Disgust (*M* = 3.89, *SD* = 2.09) positioned slightly below. All variables exhibited mild-to-moderate skewness (range: −0.92 to 0.41) and were platykurtic (kurtosis range: −1.29 to −0.36). Shapiro–Wilk tests were significant for most variables, as is typical with *N* > 200; maximum likelihood estimation in structural equation modelling is robust to such departures from normality.

### 3.3. Bivariate Correlations and Multicollinearity

The bivariate correlation matrix is presented in Table 3. Within the core TPB constructs, Attitude, Subjective Norms, and Perceived Behavioural Control were each strongly correlated with Intention (*r* = 0.694, 0.761, and 0.709, respectively; all *p* < 0.001), while Subjective Norms and Perceived Behavioural Control were also substantially correlated with each other (*r* = 0.697; *p* < 0.001). This pattern was consequential for the bootstrap stability of the SN and PBC paths in the latent SEM (Section 3.5). Among the perceptual predictors, Ethics showed a strong positive association with Attitude (*r* = 0.502; *p* < 0.001), Disgust showed a weak negative association (*r* = −0.139; *p* < 0.05), and Unnaturalness showed no significant association with Attitude (*r* = 0.055), foreshadowing the inconclusive H4 result. Unnaturalness and Disgust were themselves strongly correlated (*r* = 0.690; *p* < 0.001), motivating the multicollinearity diagnostics reported below. The Spearman coefficients (Table 3, above the diagonal) were closely convergent with the Pearson values across all variable pairs, supporting approximately linear associations and indicating that the patterns are not artefacts of the ordinal scaling of Likert items.

Variance Inflation Factors were inspected to assess multicollinearity. For the predictors of Attitude, VIFs ranged from 1.18 (Ethics) to 2.26 (Unnaturalness); for the predictors of Intention, VIFs ranged from 1.97 (PBC) to 2.64 (Subjective Norms). All values were well below the conventional threshold of 5, indicating that multicollinearity does not invalidate the regression estimates. Nevertheless, the shared variance between Subjective Norms and Perceived Behavioural Control, and between Unnaturalness and Disgust, is taken into account when the structural results are interpreted (Section 3.5).

### 3.4. Structural Model—Hypothesis Testing

The latent structural equation model exhibited mixed global fit. The incremental fit indices satisfied the conventional thresholds (CFI = 0.920; TLI = 0.901; both ≥ 0.90), but the Root Mean Square Error of Approximation exceeded the recommended cutoff (RMSEA = 0.110), indicating suboptimal absolute fit. The χ^2^ test was significant (χ^2^(77) = 313.50, *p* < 0.001), as is typical with samples of *N* > 200. The Unmeasured Latent Method Construct (ULMC) model, incorporating a latent method factor to control for common-method variance, demonstrated improved fit across all indices (CFI = 0.961; TLI = 0.945; RMSEA = 0.082; SRMR = 0.045), indicating that some shared method variance is present in the data.

**Figure 2:**
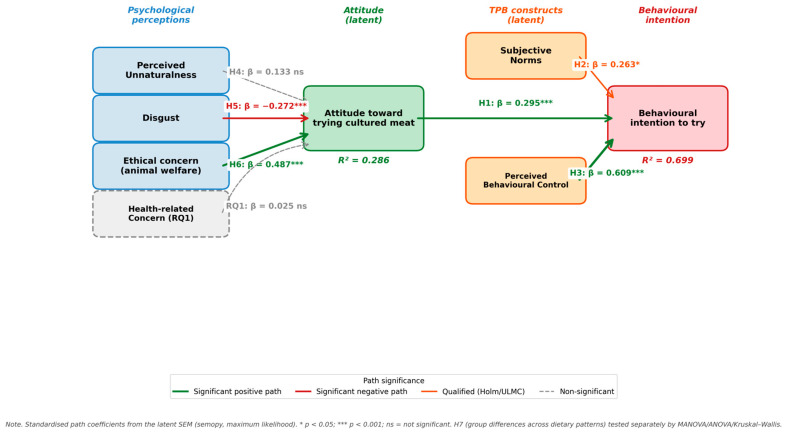
Latent structural equation model—standardised path coefficients.

Standardised path coefficients and the results of the Holm-corrected hypothesis testing are reported in Table 4 and visualised in the path diagram in Figure 2. Among the core TPB predictors of Intention (H1–H3), all three exhibited the expected positive direction. Perceived Behavioural Control was the strongest predictor of Intention (H3: β = 0.609, *p* < 0.001; Holm-corrected *p* < 0.001), followed by Attitude (H1: β = 0.295, *p* < 0.001; Holm-corrected *p* < 0.001). Subjective Norms also predicted Intention in the baseline model (H2: β = 0.263, *p* = 0.032), but the path failed to retain significance under the Holm correction (Holm-corrected *p* = 0.064) and lost significance entirely in the ULMC robustness model (β = 0.417, *p* = 0.111), indicating that the effect of subjective norms is sensitive to both multiple-testing correction and common-method variance. The bootstrap analysis (Table S2) confirmed this pattern: 25.9% of the 1000 bootstrap replicates exhibited extreme estimates for the Subjective Norms and Perceived Behavioural Control paths that exceeded the |β| = 1.5 stability threshold, reflecting the high collinearity between the two predictors (*r* = 0.697). The interpretation of H2 is therefore qualified accordingly.

Among the extended antecedents of Attitude (H4–H6) and the research question (RQ1), Ethics emerged as the strongest predictor of Attitude (H6: β = 0.487, *p* < 0.001; Holm-corrected *p* < 0.001), with the effect remaining robust in the ULMC model (β = 0.361, *p* < 0.001) and across all triangulation analyses. Disgust was a significant negative predictor (H5: β = −0.272, *p* < 0.001; Holm-corrected *p* = 0.002), although the effect was attenuated to marginal significance in the ULMC model (β = −0.174, *p* = 0.072). Perceived Unnaturalness did not significantly predict Attitude (H4: β = 0.133, *p* = 0.103; Holm-corrected *p* = 0.103) and, unexpectedly, had a positive rather than the hypothesised negative direction. The exploratory predictor Health Concern was likewise non-significant (RQ1: β = 0.025, *p* = 0.729; BH-FDR-corrected *p* = 0.729). Hypotheses H1, H3, H5, and H6 were therefore supported under both raw and corrected *p*-values; H2 was supported in the baseline model only; H4 and RQ1 were not supported. The two endogenous constructs were well explained by their predictors: *R*^2^ = 0.699 for Intention and *R*^2^ = 0.286 for Attitude (see also the path diagram in Figure 2 for the full set of estimated paths).

### 3.5. Robustness and Triangulation Analyses

**Figure 3:**
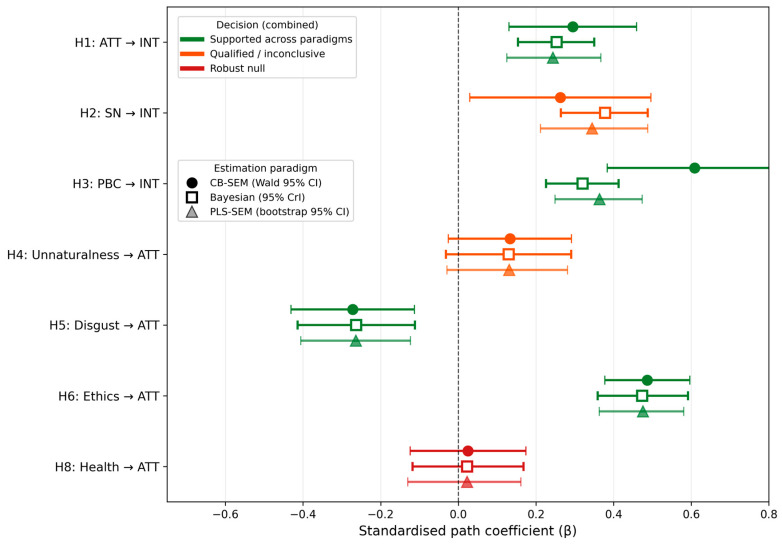
Multi-paradigm triangulation of structural path estimates.

**Figure 4:**
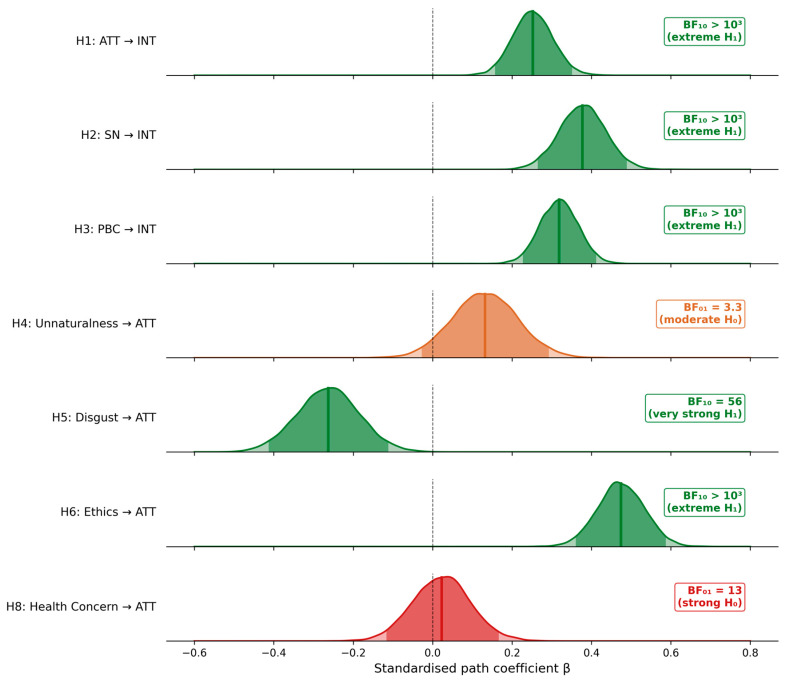
Bayesian path model—posterior densities and Savage–Dickey Bayes factors.

The convergence of results across the four estimation paradigms is summarised in Table 5 and visualised as a forest plot in Figure 3, providing strong triangulation evidence for the structural conclusions. The Bayesian path model yielded posterior means closely aligned with the frequentist estimates (e.g., H1 posterior mean = 0.253, 95% credible interval [0.154, 0.350]; H6 posterior mean = 0.474, 95% CrI [0.359, 0.592]). The posterior probability of direction exceeded 0.99 for H1, H2, H3, H5, and H6, supporting the corresponding decisions. The Savage–Dickey approximations of the Bayes factor against a point null were extremely large for the supported paths (*BF*_10_ > 10^5^ for H1, H2, H3, H6) and identified two qualitatively different evidentiary patterns among the non-significant predictors: for H4 (Unnaturalness), the Bayes factor provided moderate evidence in favour of the null (*BF*_01_ = 3.3), whereas for RQ1 (Health Concern), the evidence in favour of the null was strong (*BF*_01_ = 13). This distinction is particularly informative for the interpretation of the two null findings; the full set of posterior densities, with the corresponding Bayes factors, is shown in Figure 4; the MCMC convergence diagnostics are reported in Table S7.

**Figure 5:**
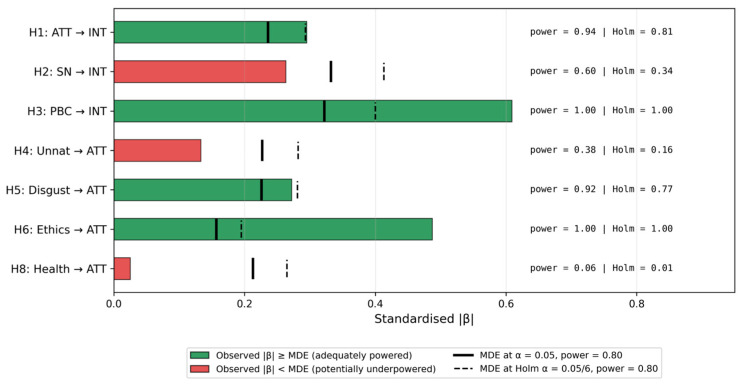
Sensitivity power analysis—observed effect vs. minimum detectable effect.

The post hoc sensitivity power analysis, summarised in Table 6 and visualised in Figure 5 (full per-path results in Table S3), reinforces this distinction. Power to detect the observed Unnaturalness–Attitude path at the uncorrected α = 0.05 was estimated at 0.38, with a minimum detectable standardised effect of 0.227 at power = 0.80, a value substantially larger than the observed β = 0.133. The non-significance of H4 may therefore reflect insufficient power rather than a true null effect. By contrast, power to detect the RQ1 effect was estimated at 0.06 with an observed β of 0.025; this combination—convergent Bayesian support for the null, a minute observed effect, and very low power for detection—indicates that RQ1 represents a substantively negligible effect rather than an underpowered non-detection. Power was adequate (≥0.80) for the supported paths H1, H3, H5, and H6 at both the uncorrected and Holm-corrected α thresholds.

The relative-importance ranking of the predictors, quantified by Budescu’s general dominance decomposition, is reported in Table 6. For the prediction of Intention, Subjective Norms contributed 38.8% of the explained variance, Perceived Behavioural Control 32.8%, and Attitude 28.4%. This ordering differs from the ranking implied by the standardised SEM path coefficients, in which Perceived Behavioural Control exhibits the largest unique partial contribution, and reflects the high shared variance among the three TPB predictors. For the prediction of Attitude, Ethics dominated the explained variance (79.3%), with Disgust accounting for 13.2% and Unnaturalness and Health Concern accounting for negligible proportions (3.1% and 4.3%, respectively). The dominance ranking complements the partial-regression evidence for the H1–H3 predictors of Intention and for the H4–H6 plus RQ1 predictors of Attitude.

The binary-outcome sensitivity analysis provided additional support for the core TPB structure. When Intention was dichotomised at the scale midpoint, a logistic regression of the dichotomised outcome on the three core TPB predictors (with HC3 robust standard errors) yielded *AUC* = 0.927 and McFadden’s pseudo-*R*^2^ = 0.493 (Table S4a), indicating very strong discrimination between respondents willing and unwilling to try cultured meat. The corresponding model for dichotomised Attitude—regressed on the four perceptual predictors—yielded McFadden’s pseudo-*R*^2^ = 0.187 (Table S4b), consistent with the more modest explanatory power of the perceptual predictors in the SEM (*R*^2^ = 0.286).

The PLS-SEM analysis (Table S6a,b), the elastic net regression (Table S5a), and the random forest analysis (Table S5b) all converged on the same predictor set. The PLS-SEM path estimates were closely aligned with the covariance-based SEM estimates for the antecedents of Attitude (H4: β = 0.131 vs. 0.133; H5: β = −0.264 vs. −0.272; H6: β = 0.476 vs. 0.487; RQ1: β = 0.023 vs. 0.025; all within 0.02 of the CB-SEM estimate), and the elastic net regression excluded both Unnaturalness and Health Concern as predictors of Attitude, consistent with the null findings for H4 and RQ1 in the SEM. The random forest permutation importance reproduced the dominance-analysis ordering (for Intention: Subjective Norms > Perceived Behavioural Control > Attitude; for Attitude: Ethics ≫ Disgust > Unnaturalness > Health Concern). The cross-validated *R*^2^ values from the elastic net (Intention: 0.697; Attitude: 0.272) corresponded closely with the SEM-derived *R*^2^ values, indicating that the structural conclusions are not contingent on the choice of estimation paradigm.

Two further sensitivity analyses addressed the measurement and collinearity concerns surrounding Perceived Behavioural Control. First, to assess the sensitivity of the PBC effect to its two-item parameterisation, the latent model was re-estimated with PBC represented by each indicator alone (Table S9). The PBC → Intention path remained positive and significant in all three specifications (two-indicator β = 0.609; E1-only β = 0.552; E2-only β = 0.138; all *p* < 0.01), and the Attitude and Subjective Norms paths retained their direction and significance throughout, indicating that the core structural conclusions do not depend on the two-item operationalisation. However, the magnitude of the PBC effect was substantially larger when PBC was indexed by the accessibility item E1 (“I could easily try cultured meat once it becomes available”) than by the autonomy item E2 (“whether I try cultured meat is entirely up to me”), suggesting that the predictive weight of PBC in this sample is carried primarily by perceived market accessibility rather than by personal autonomy. Second, an alternative hierarchical model in which Subjective Norms was specified as an antecedent of PBC (SN → PBC → Intention) rather than as a parallel predictor fitted the data equally well (CFI = 0.920; TLI = 0.902; RMSEA = 0.110) and was marginally preferred by the information criteria (AIC = 51.5 versus 53.5; BIC = 147.1 versus 152.7; Table S10). In this model, the SN → PBC path was very strong (β = 0.865, *p* < 0.001) and the PBC → Intention path strengthened further (β = 0.859, *p* < 0.001), consistent with Ajzen’s argument that social pressures shape perceived behavioural control and offering a theoretically coherent resolution of the high SN–PBC collinearity (*r* = 0.697) observed in the parallel model.

### 3.6. Mediation Analysis

Bootstrap mediation analysis (5000 resamples; percentile confidence intervals) examined whether the four perceptual predictors exert indirect effects on Intention via Attitude. Two indirect pathways were significant. The indirect effect of Ethics on Intention via Attitude was positive and was the largest of the four (ab = 0.114; 95% CI [0.056, 0.183]), indicating that ethical concern for animal welfare enhances favourable attitudes, which in turn strengthen behavioural intention. The indirect effect of Disgust on Intention via Attitude was negative and significant (ab = −0.063; 95% CI [−0.122, −0.019]), indicating that affective aversion reduces favourable attitudes and consequently reduces intention. The indirect effects of Unnaturalness (ab = 0.030; 95% CI [−0.007, 0.076]) and Health Concern (ab = 0.006; 95% CI [−0.033, 0.048]) on Intention via Attitude were non-significant. As none of the four perceptual predictors exhibited significant direct effects on Intention (all *p* > 0.28 in the partial mediation model), the effects of Ethics and Disgust on Intention were fully mediated through Attitude. Given the cross-sectional design, these results are interpreted as indirect associations rather than as evidence of causal mediation.

### 3.7. Group Differences by Dietary Pattern

Hypothesis H7, which posited that Attitude and Intention differ across dietary patterns, was not supported. A multivariate analysis of variance on the joint vector of Attitude and Intention across the three larger dietary groups (Omnivore, Flexitarian, and Vegetarian; vegans excluded owing to insufficient sample size) was non-significant according to Pillai’s Trace (V = 0.012; *F*(4, 496) = 0.74; *p* = 0.567) and the corroborative Wilks’ Lambda (Λ = 0.988; *F*(4, 494) = 0.73; *p* = 0.569). The univariate ANOVAs were similarly non-significant for both outcomes (Attitude: *F*(2, 248) = 0.43, *p* = 0.654, η^2^ = 0.003, ω^2^ = 0.000, Cohen’s f = 0.059; Intention: *F*(2, 248) = 0.78, *p* = 0.461, η^2^ = 0.006, ω^2^ = 0.000, Cohen’s f = 0.079). The non-parametric Kruskal–Wallis tests, including all four dietary groups, also yielded non-significant results, although the test for Attitude approached conventional significance (*H*(3) = 7.33, *p* = 0.062, ε^2^ = 0.017; full MANOVA results in Table S8a and univariate ANOVA/Kruskal–Wallis results in Table S8b). All effect sizes were negligible (η^2^ < 0.01). H7 was therefore not supported under any analytic approach.

### 3.8. Summary of Hypothesis Testing

The integrated decision pattern across all analytic layers is reported in Table 5 (Panel B) and is summarised as follows. Four of the seven hypotheses and the research question were robustly supported across the latent SEM with Holm-corrected *p*-values, the bootstrap robust inference, the ULMC robustness check, the Bayesian path model, and the PLS-SEM and machine learning triangulation: H1 (Attitude → Intention), H3 (Perceived Behavioural Control → Intention), H5 (Disgust → Attitude), and H6 (Ethics → Attitude). The decision for H2 (Subjective Norms → Intention) is qualified: the baseline path was significant (*p* = 0.032) but failed to retain significance under Holm correction (*p* = 0.064) and under common-method-variance control (*p* = 0.111). H4 (Unnaturalness → Attitude) was not supported in the latent SEM but was found to be underpowered (estimated power = 0.38) and the Bayes factor evidence for the null was moderate (*BF*_01_ = 3.3); H4 is therefore best characterised as inconclusive rather than as evidence of absence. RQ1 (Health Concern → Attitude) was robustly non-significant in the SEM, and the Bayesian analysis provided strong evidence in favour of the null (*BF*_01_ = 13); RQ1 is therefore characterised as a substantive null finding. H7 (group differences across dietary patterns) was not supported under any analytic approach, with negligible effect sizes throughout.

## 4. Discussion

### 4.1. Overview of Findings

The present study applied an extended Theory of Planned Behaviour (TPB) framework, complemented by a pre-specified package of robustness and triangulation analyses, to examine the psychological predictors of cultured-meat acceptance among English-speaking Indian consumers (*N* = 255). Across covariance-based structural equation modelling, Bayesian path modelling, PLS-SEM, and regularised machine learning estimation, a coherent pattern emerged: Perceived Behavioural Control and ethical concern for animal welfare were the strongest predictors of acceptance, disgust constituted a significant emotional barrier, and the conventional Western emphasis on perceived unnaturalness was not supported in the present sample (with the H4 result further characterised as inconclusive rather than as a substantive null, on the basis of the sensitivity power analysis and Bayes-factor evidence; see Section 4.3). Across the seven hypotheses and one research question, four hypotheses were robustly supported in every analytic layer (H1, H3, H5, and H6); one hypothesis was supported in the baseline model but failed to retain significance under both Holm–Bonferroni correction and common-method-variance control (H2); one hypothesis was inconclusive owing to insufficient power (H4); one hypothesis received no support (H7); and the research question (RQ1) was characterised as a substantively supported null on the basis of strong Bayesian evidence in favour of the null (*BF*_01_ = 13). The findings extend the TPB literature into a previously underrepresented context and complicate the dominant Western narrative about which psychological mechanisms govern cultured-meat acceptance. They also complement recent cross-cultural evidence on consumer trust in emerging food technologies in India [23] by specifying the structural pathway—dominated by perceived feasibility and animal-welfare concern—through which such perceptions translate into the intention to try cultured meat.

### 4.2. Core TPB Findings: The Prominence of Perceived Behavioural Control

The three core TPB predictors of intention were directionally consistent with the established literature but exhibited a relative-importance pattern that departs from typical Western findings. Perceived Behavioural Control emerged as the strongest standardised predictor of Intention (β = 0.609, *p* < 0.001), accounting for the largest unique contribution within the structural equation model. This primacy is, however, partial: it reflects unique variance after the contributions of the other predictors have been controlled. As the dominance analysis shows (Section 4.5), Subjective Norms commands the largest share of total explained variance when shared variance is preserved. The claim of PBC primacy is therefore confined to the partial-regression perspective. This is in marked contrast to recent extended-TPB studies of cultured-meat acceptance in Germany, in which Attitude has been reported as the strongest predictor and Perceived Behavioural Control as a secondary predictor [11,12]. A consistent pattern was reported by Malavalli et al. [42] for New Zealand respondents, where PBC was a robust predictor of intention under conditions of low product familiarity. The present results therefore align with the broader observation that, in early-stage acceptance research for novel food technologies, perceived feasibility tends to dominate over evaluative attitude when the product is not yet available in the market.

Several mechanisms may account for the prominence of Perceived Behavioural Control in the Indian context. First, cultured meat has no commercial presence in India at the time of writing, and only 45.1% of the present sample reported any prior awareness of the technology. Under such conditions of high novelty and low market availability, behavioural intention may be primarily contingent on whether the act of trying the product is perceived as feasible—that is, on perceived accessibility, affordability, and personal autonomy—rather than on the strength of attitudinal evaluation, which is itself only weakly formed in the absence of direct sensory experience [3]. Second, the structural-model decomposition reflects the partial regression coefficient, which captures the unique contribution of a predictor after the variance shared with the other predictors has been removed. As the dominance analysis revealed (see Section 4.5), the relative-importance ranking of the three TPB predictors is substantially altered when shared variance is preserved.

Attitude exhibited a smaller but robust positive effect on Intention (H1: β = 0.295, *p* < 0.001; Holm-corrected *p* < 0.001; Bayesian posterior mean = 0.253, *BF*_10_ > 10^3^). The role of Subjective Norms was more equivocal. The baseline model reported a significant positive effect of Subjective Norms on Intention (H2: β = 0.263, *p* = 0.032), but the path failed to retain significance under the Holm–Bonferroni correction (*p* = 0.064) and was further attenuated to non-significance in the ULMC robustness check (β = 0.417, *p* = 0.111). The non-parametric bootstrap, in which approximately one quarter of replicates produced extreme estimates for the Subjective Norms and Perceived Behavioural Control paths, provided independent evidence that the two predictors are partially confounded in the present sample (r = 0.697). The role of social norms in Indian cultured-meat acceptance therefore remains an open question. Indian dietary identity and religious dietary codes might be expected a priori to amplify the role of social norms, yet the present evidence is sensitive to multiple analytic decisions and does not warrant a firm conclusion. Recent work shows that even within Western samples, vegetarians and omnivores diverge in how they categorise and evaluate cultured meat [43], suggesting that dietary identity may shape acceptance in ways not captured by aggregate group comparisons.

### 4.3. Two Qualitatively Different Null Findings

One analytical contribution of this study to the cultured-meat acceptance literature is the explicit identification of two non-significant paths that are typically conflated under a single “not supported” decision but that are qualitatively different in their evidentiary status: H4 (Perceived Unnaturalness → Attitude) and RQ1 (Health-related Concern → Attitude).

For H4, the observed effect was small and positive (β = 0.133), rather than in the hypothesised negative direction, and it was not significant under either raw or Holm-corrected *p*-values. However, the sensitivity power analysis revealed that the post hoc power for detecting this effect was only 0.38 at the uncorrected α = 0.05 and 0.16 at the Holm-corrected α = 0.05/6; the minimum detectable standardised effect at conventional power was 0.227, substantially larger than the observed β. The Bayesian Savage–Dickey analysis returned moderate evidence in favour of the null (*BF*_01_ = 3.3). The combination of low statistical power, an observed effect below the minimum detectable threshold, and modest Bayesian evidence for the null indicates that the non-significant H4 result is best characterised as inconclusive rather than as evidence of a true null. Replication with substantially larger samples—approximately *N* = 500–600 by the present power estimates—would be required to determine whether perceived unnaturalness exerts a small but real influence on attitudes in the Indian context. The H4 result therefore warrants caution: it should not be interpreted as evidence that perceived unnaturalness is irrelevant to cultured-meat acceptance in India, but only as evidence that the present study was not adequately powered to detect an effect of the observed magnitude. Recent cross-food evidence likewise indicates that perceived unnaturalness and disgust function as coupled yet culturally variable barriers across cultured foods [44]. Consistent with the inconclusive characterisation of H4 in the present sample, a recent cross-national European study by Loera et al. [45] of consumers in Italy, France, and the Netherlands (*N* = 1500) reported that the natural-is-better heuristic—the conceptual cousin of perceived unnaturalness—did not significantly predict intention to try cultured meat once trust in food safety authorities and food technophobia were controlled. The convergence with the present non-significant H4 result across two independent non-overlapping samples reinforces the empirical pattern, even while the interpretive distinction between an inconclusive non-significance (the present result) and a substantively supported null nevertheless remains relevant to the cultured-meat acceptance literature.

The interpretation of RQ1 is fundamentally different. The observed effect of Health-related Concern on Attitude was virtually zero (β = 0.025), the post hoc power was negligible (0.06), and the Bayesian analysis returned strong evidence in favour of the null (*BF*_01_ = 13). This combination—a near-zero point estimate, near-zero detection probability, and strong Bayesian support for the null—provides much firmer grounds for the substantive conclusion that, in the present sample, health-related concern does not act as a meaningful independent predictor of attitudes toward cultured meat after the variance shared with disgust and ethical concern is accounted for. The implication is not that Indian consumers do not have health-related concerns about cultured meat; on the contrary, the mean health-concern score was the highest of all measured variables (M = 5.39 on a 7-point scale). Rather, the implication is that such concerns operate through, or are subsumed by, the affective dimension of disgust and the moral dimension of ethical evaluation, rather than acting as an independent cognitive antecedent of attitude formation. This pattern is consistent with the conceptual argument advanced by Wilks, Hornsey, and Bloom [46], who note that lay perceptions of “unnaturalness” and “health risk” in novel foods tend to converge with affective and moral evaluations rather than constituting separable cognitive antecedents.

The distinction between an inconclusive non-significance (H4) and a substantively supported null (RQ1) is rarely articulated in the cultured-meat acceptance literature, in which the conventional reporting framework subsumes both outcomes under the single decision “hypothesis not supported.” The Bayesian Savage–Dickey approach [47,48] provides a formal mechanism for separating these two outcomes and is recommended for future research in this domain.

### 4.4. Ethics, Ahimsa, and the Moral Foundation of Purity

Before interpreting the Ethics effect (H6) in cultural terms, it must be stated explicitly that neither ahimsa endorsement nor purity-based moral reasoning was directly operationalised in the questionnaire. The cultural mechanisms proposed in this section are therefore theoretical propositions advanced for future empirical testing, rather than constructs measured in the present study, and they are framed accordingly. The strongest predictor of Attitude in the present study was ethical concern for animal welfare (H6: β = 0.487, *p* < 0.001; Bayesian posterior mean = 0.474, *BF*_10_ > 10^3^), and this effect remained robust under every robustness and triangulation check. The dominance analysis indicated that ethical concern accounted for 79.3% of the variance in Attitude explained jointly by the four perceptual predictors. The magnitude and robustness of this effect deserve contextual interpretation that goes beyond the standard observation that animal-welfare framing predicts cultured-meat acceptance [2,9].

The principle of ahimsa—non-violence toward all sentient beings—is foundational to Hindu and Jain ethical traditions and is institutionalised in Indian dietary practice through India’s large vegetarian population, the legally mandated green/brown dot food-labelling system that classifies packaged products as vegetarian or non-vegetarian, and religious dietary codes such as halal [49,50]. Cultured meat is conceptually unusual because it is biologically meat yet does not require the slaughter of a live animal. This places it in an ambiguous position relative to the ahimsa framework but also allows it to be framed as a means of reducing rather than perpetuating animal suffering. The strong association observed in the present sample between ethical concern for animal welfare and Attitude is consistent with the hypothesis that respondents who hold ahimsa-aligned values may evaluate cultured meat through that ethical lens. Ahimsa itself was not directly measured in the present study, however, and the ahimsa interpretation therefore remains theoretically motivated rather than empirically tested. Comparable evidence of the centrality of ethical reasoning in South Asian acceptance has been reported by Ahsan, Uzair, and Ali [51] in their study of Pakistani consumers.

A particularly relevant theoretical lens has recently been advanced by Wilks, Crimston, and Hornsey [10], who demonstrated that the moral foundation of purity—rather than harm—is the strongest moral predictor of attitudes toward cultured meat in cross-cultural samples. The purity-pollution distinction is deeply embedded in Hindu and Jain food traditions through the concept of shuddhi (ritual purity) and through caste-related dietary practices. The Indian context therefore provides a particularly informative setting in which to test the purity-moral-foundation hypothesis. Future research that directly measures purity-based moral reasoning in Indian samples is necessary to determine whether the observed ethics effect is mediated by, or conceptually distinct from, purity-based moral evaluation. The cultural-theoretical considerations advanced in this section should be regarded as candidate interpretive frameworks whose validity awaits direct empirical testing in the Indian context; the present study did not measure ahimsa endorsement, purity-based moral reasoning, or shuddhi-related dietary practices, and the manuscript does not claim to have demonstrated that any of these cultural constructs mediates the observed Ethics effect.

Disgust exhibited the expected significant negative effect on Attitude (H5: β = −0.272, *p* < 0.001), although the effect was attenuated to marginal significance under ULMC control (β = −0.174, *p* = 0.072), suggesting partial sensitivity to shared-method variance. The mediation analysis revealed that both the ethical effect (H6) and the disgust effect (H5) operate on Intention entirely through Attitude; neither perceptual predictor exhibited a significant direct effect on Intention. The asymmetry between the strong positive effect of Ethics (β = 0.487) and the smaller negative effect of Disgust (β = −0.272) is consistent with the argument by Rosenfeld and Tomiyama [52] and by Arango, Septianto, and Pontes [53] that positive ethical framing may be a more potent lever for shifting attitudes than reducing affective aversion.

### 4.5. Practical Implications: Dominance Versus Partial Regression

The dominance analysis revealed a relative-importance ranking that differs from the ranking implied by the standardised SEM path coefficients and that has different practical implications. Within the SEM, the standardised partial regression coefficients yielded the ranking PBC > Attitude > Subjective Norms (β = 0.609, 0.295, 0.263) for the prediction of Intention. The general dominance decomposition [40,41], which is order-independent and preserves rather than partials out shared variance, yielded a different ranking: Subjective Norms (38.8% of the explained variance) > PBC (32.8%) > Attitude (28.4%). The two rankings reflect different but complementary perspectives on the relative importance of the TPB predictors.

For marketing and communication interventions, the dominance result suggests that targeting Subjective Norms—through influencer endorsements, peer-to-peer testimonials, or culturally credible spokespersons—may have the greatest leverage on intention to try cultured meat in the Indian market, because Subjective Norms carries the largest share of the explained variance when the overlap with PBC and Attitude is preserved. For policy and regulation, the dominance of Perceived Behavioural Control within the SEM (β = 0.609) signals that improving the perceived accessibility, affordability, and autonomy of cultured meat—through product availability, transparent pricing, and clear labelling—remains essential, because PBC carries the largest unique contribution after social and attitudinal influences are accounted for. The two analytic perspectives are therefore complementary rather than contradictory: dominance analysis identifies the largest overall lever, while standardised regression identifies the largest unique lever. A coordinated Indian market strategy will benefit from interventions on both dimensions, with social-norm campaigns and accessibility-focused policy operating in parallel.

For India’s substantial Muslim consumer population, halal certification adds an additional regulatory dimension. Hamdan et al. [54] provided empirical evidence that the willingness of Muslim consumers to substitute cultured meat for conventionally slaughtered meat is conditional on credible halal certification from recognised religious authorities. The halal-certification framework for cultured meat is therefore a practical consumer-acceptance issue rather than a purely theoretical concern, and it warrants explicit policy attention as the Indian regulatory landscape for cultivated proteins matures.

### 4.6. Methodological Contributions and Limitations

The present study contributes to the cultured-meat acceptance literature on three distinct levels. Empirically, it provides a theory-driven analysis of TPB predictors in an English-speaking Indian sample, addressing the geographic gap noted in recent reviews [4,5]. Theoretically, it positions cultured meat against the ahimsa framework and articulates how a moral-foundation lens—particularly purity rather than harm [10]—might structure acceptance in a non-Western context, thereby motivating the direct measurement of these constructs in future work. Methodologically, it combines a Holm–Bonferroni multiplicity-corrected SEM with ULMC robustness checking, non-parametric bootstrap inference, post hoc sensitivity power analysis, and triangulation across four estimation paradigms: covariance-based SEM, Bayesian path modelling, PLS-SEM [55,56], and regularised machine learning estimation. The convergence of structural conclusions across these paradigms reduces the likelihood that the findings are artefacts of any single methodological choice. Two specific methodological features warrant particular attention.

First, the explicit distinction between H4 and RQ1 as qualitatively different null findings—distinguishable by the joint pattern of observed effect size, statistical power, and Bayesian evidence ratio—represents a methodological refinement that, to our knowledge, has not previously been applied within the cultured-meat acceptance literature, where non-significance has typically been conflated with substantive null support. The Bayesian Savage–Dickey approach provides a formal mechanism for distinguishing inconclusive non-significance from substantive null evidence and is recommended for future studies in this domain.

Second, the bootstrap analysis revealed that approximately 26% of replicates produced extreme estimates for the Subjective Norms and Perceived Behavioural Control paths, reflecting the high collinearity between these predictors in the present sample. This finding constitutes empirical evidence that the partial regression coefficients for SN and PBC are weakly identified in convenience samples of the present size and structure, and it tempers any strong claims about the relative importance of these two TPB constructs. An alternative hierarchical specification in which Subjective Norms is modelled as an antecedent of Perceived Behavioural Control (Section 3.5) fitted the data equally well and offers a theoretically grounded way to represent this entanglement in future confirmatory work.

Several limitations should be acknowledged. The convenience-sample design and the requirement of English-language proficiency restrict generalisability to urban, educated, English-literate Indian adults; the present sample is best characterised as an early-adopter segment rather than as a representative cross-section of Indian consumers. The cross-sectional design precludes causal inference; the indirect effects reported in the mediation analysis are best interpreted as indirect associations rather than as evidence of causal mediation. The four perceptual predictors (Unnaturalness, Disgust, Ethics, and Health Concern) were measured by single items, which precludes formal reliability estimation, may attenuate the corresponding path estimates through unmodelled measurement error, and may constitute construct under-representation for theoretically complex constructs such as ethical concern for animal welfare. Multi-item validated scales would strengthen construct validity in future replications [9]. The Perceived Behavioural Control, Subjective Norms, and Intention scales each comprise only two indicators, which restricts the precision with which their effects can be estimated; PBC reliability was essentially at the conventional 0.70 threshold (α = 0.698; ω = 0.699; inter-item r = 0.536). Common-method variance, although controlled by the ULMC robustness check [35,36], remains a residual concern for any single-source self-report study. Finally, the present design did not directly measure several culturally specific variables—ahimsa endorsement, purity-based moral reasoning [10], halal-certification importance, and caste-based food practices—that may be operative in the Indian context; integrating such measures in future work would substantially deepen the cultural interpretation of the results.

### 4.7. Directions for Future Research

Three principal directions for future research follow from the present findings and their limitations. First, replication with probability-based samples that include rural residents, non-English speakers, and lower-educational-attainment groups is needed to extend the generalisability of the present results. The sensitivity power analysis indicates that approximately *N* = 500–600 would be required to definitively resolve the inconclusive H4 result, and substantially larger oversampled subgroups would be required to enable robust within-group comparisons across dietary identities (cf. H7). Second, integrating culturally specific measures—direct ahimsa endorsement, purity-based moral reasoning [10], halal-certification importance [54], and caste-based dietary practices—would permit a richer empirical test of the cultural-mechanism hypotheses proposed in the present discussion. Third, longitudinal and experimental designs that track attitude and intention as awareness increases and as actual product availability emerges in the Indian market are needed to assess the stability of the early-adopter patterns reported here. Field studies measuring actual purchase and consumption behaviour, rather than self-reported intention, would address the well-documented intention–behaviour gap [57] and would convert the current associational evidence into direct evidence of consumer adoption.

## 5. Conclusions

The present study provides an extended Theory of Planned Behaviour analysis of cultured-meat acceptance in India, complemented by a triangulated package of robustness analyses spanning multiplicity correction, bootstrap robust inference, post hoc sensitivity power analysis, Bayesian path modelling, PLS-SEM, and regularised machine learning estimation. Three substantive conclusions follow.

First, the psychological architecture of cultured-meat acceptance among Indian consumers is dominated by perceived feasibility and by ethical concern for animal welfare. Perceived Behavioural Control was the strongest predictor of behavioural intention (β = 0.609), and ethical concern for animal welfare was the strongest predictor of attitude (β = 0.487). Disgust functioned as a significant emotional barrier (β = −0.272). These three effects were robust across all four estimation paradigms and across the full set of robustness checks. The Indian pattern therefore differs from the attitude-dominant pattern reported in recent Western extended-TPB studies and suggests that, in the early-adoption phase of a novel food technology with no current market presence, perceived feasibility and moral evaluation are the principal levers.

Second, two non-significant findings—perceived unnaturalness (H4) and health-related concern (RQ1)—are qualitatively distinct in their evidentiary status. The H4 result was inconclusive: the observed effect lay below the minimum detectable effect at conventional power, and the Bayesian evidence in favour of the null was only moderate. The RQ1 result, by contrast, was supported by strong Bayesian evidence in favour of the null (*BF*_01_ = 13). The conventional reporting framework that subsumes both outcomes under a single “hypothesis not supported” decision is therefore insufficient; the joint use of frequentist significance testing, power analysis, and Bayesian evidence ratios provides a more nuanced evidentiary scheme that should be standard in future cultured-meat acceptance research.

Third, the practical implications for introducing cultured meat to the Indian market follow from the joint interpretation of partial-regression and dominance evidence. Standardised SEM coefficients identify Perceived Behavioural Control as the largest unique predictor of intention, suggesting that accessibility, affordability, and clear labelling are essential policy levers. The dominance decomposition identifies Subjective Norms as the largest overall contributor to explained variance, suggesting that peer endorsement, influencer communication, and culturally credible spokespersons are essential marketing levers. The two perspectives are complementary, and a coherent Indian cultured-meat strategy will integrate both. Animal-welfare framing, communicated in a culturally sensitive way and tested in future studies against constructs such as ahimsa endorsement, purity-based moral reasoning, and halal-certification importance, may constitute an important component of future Indian cultured-meat communication strategies. Halal certification for the substantial Muslim consumer population and the gradual reduction of affective aversion through repeated exposure are also likely to be important tactical components of such a strategy. The findings position India as a distinctive but tractable market for the global protein transition.

## Figures and Tables

**Table 1:** Sample characteristics (*N* = 255).

Characteristic	Category	*n*	%
Gender	Male	177	69.4
	Female	73	28.6
	Prefer not to disclose	5	2.0
Education	Bachelor’s degree	143	56.1
	Master’s degree or higher	102	40.0
	High school	7	2.7
	Vocational/technical	2	0.8
	Primary or lower secondary	1	0.4
Place of residence	City > 500,000	108	42.4
	City 100,000–500,000	62	24.3
	City 20,000–100,000	35	13.7
	Rural area	30	11.8
	Town < 20,000	20	7.8
Dietary pattern	Omnivore	161	63.1
	Flexitarian (limits meat consumption)	73	28.6
	Vegetarian	17	6.7
	Vegan	4	1.6
Prior awareness of cultured meat	Yes	115	45.1
	No	140	54.9

Note. All respondents reported India as their country of residence.

**Table 2:** Descriptive statistics and scale reliability for all study variables (*N* = 255).

Variable	k Items	*M*	*SD*	Skew	Kurt	α	ω	Inter-Item *r*
Attitude (ATT)	4	3.36	1.84	0.41	−0.97	0.949	0.950	—
Subjective Norms (SN)	2	3.47	2.01	0.38	−1.06	0.934	0.936	0.876
Perceived Behavioural Control (PBC)	2	4.20	1.93	−0.17	−1.04	0.698	0.699	0.536
Intention (INT)	2	3.57	2.10	0.24	−1.29	0.908	0.909	0.831
Unnaturalness	1	4.59	2.16	−0.34	−1.26	—	—	—
Disgust	1	3.89	2.09	0.11	−1.24	—	—	—
Ethics	1	4.57	2.08	−0.37	−1.12	—	—	—
Health Concern	1	5.39	1.91	−0.92	−0.36	—	—	—

Note. *M* = mean; *SD* = standard deviation; Skew = skewness; Kurt = excess kurtosis; α = Cronbach’s alpha; ω = McDonald’s omega total estimated via confirmatory factor analysis loadings; Inter-item *r* = Pearson correlation between the two items (reported for two-item scales only). Single-item measures (k = 1) do not permit reliability estimation. All scales are scored on 7-point Likert (or semantic-differential, for Attitude) responses.

**Table 3:** Bivariate correlations: Pearson r below the diagonal, Spearman ρ above the diagonal (*N* = 255).

	ATT	SN	PBC	INT	Unnat	Disg	Ethics	HC
ATT	—	0.687	0.553	0.696	0.035	−0.138	0.494	0.055
SN	0.689 ***	—	0.691	0.772	0.028	−0.137	0.438	0.061
PBC	0.552 ***	0.697 ***	—	0.714	0.248	0.034	0.467	0.298
INT	0.694 ***	0.761 ***	0.709 ***	—	0.076	−0.117	0.443	0.108
Unnat	0.055	0.071	0.285 ***	0.118	—	0.699	0.193	0.590
Disg	−0.139 *	−0.120	0.042	−0.105	0.690 ***	—	0.034	0.509
Ethics	0.502 ***	0.439 ***	0.449 ***	0.461 ***	0.184 **	0.042	—	0.327
HC	0.123 *	0.129 *	0.374 ***	0.191 **	0.604 ***	0.533 ***	0.333 ***	—

Note. ATT = Attitude; SN = Subjective Norms; PBC = Perceived Behavioural Control; INT = Intention; Unnat = Unnaturalness; Disg = Disgust; HC = Health Concern. Below the diagonal: Pearson product-moment correlations. Above the diagonal: Spearman rank-order correlations. Significance stars apply to the Pearson coefficients. * *p* < 0.05, ** *p* < 0.01, *** *p* < 0.001.

**Table 4:** Latent structural model—path coefficients with Holm–Bonferroni multiplicity correction and common-method-variance (ULMC) robustness check (*N* = 255).

Hyp.	Path	β	*p*	Holm *p*	β (ULMC)	*p* (ULMC)
H1	Attitude → Intention	0.295	<0.001	<0.001	0.370	<0.001
H2	Subjective Norms → Intention	0.263	0.032	0.064	0.417	0.111
H3	PBC → Intention	0.609	<0.001	<0.001	0.558	<0.001
H4	Unnaturalness → Attitude	0.133	0.103	0.103	0.078	0.433
H5	Disgust → Attitude	−0.272	<0.001	0.002	−0.174	0.072
H6	Ethics → Attitude	0.487	<0.001	<0.001	0.361	<0.001
RQ1	Health Concern → Attitude	0.025	0.729	0.729	0.002	0.985

Note. β = standardised structural path coefficient. *p* = raw two-sided Wald *p*-value. Holm *p* = Holm–Bonferroni adjusted *p*-value for the H1–H6 family at α = 0.05; Benjamini–Hochberg q-value reported for RQ1. β (ULMC) and *p* (ULMC) = coefficient and *p*-value in the model including an unmeasured latent method construct.

**Table 5:** Multi-paradigm triangulation of structural path estimates and integrated decision across estimation paradigms (*N* = 255).

Panel A. Multi-paradigm triangulation: Bayesian path model, PLS-SEM, and bootstrap inference.						
Hyp.	Path	Bayes mean	95% CrI	*BF*_10_	PLS β	Bootstrap 95% CI
H1	Attitude → Intention	+0.253	[+0.154, +0.350]	very large	+0.244	[+0.142, +0.463]
H2	Subjective Norms → Intention	+0.378	[+0.264, +0.488]	very large	+0.345	[+0.056, +0.541]
H3	PBC → Intention	+0.320	[+0.226, +0.413]	very large	+0.364	[+0.344, +0.778]
H4	Unnaturalness → Attitude	+0.130	[−0.032, +0.291]	0.30 (*BF*_01_ = 3.3)	+0.131	[−0.029, +0.292]
H5	Disgust → Attitude	−0.263	[−0.414, −0.112]	55.8	−0.264	[−0.430, −0.110]
H6	Ethics → Attitude	+0.474	[+0.359, +0.592]	very large	+0.476	[+0.374, +0.593]
RQ1	Health Concern → Attitude	+0.023	[−0.118, +0.168]	0.07 (*BF*_01_ = 14.3)	+0.023	[−0.131, +0.170]
Panel B. Integrated decision across all analytic layers.						
Hypothesis	Path		Final Decision			
H1	Attitude → Intention		Supported			
H2	Subjective Norms → Intention		Supported in baseline; not retained under Holm or ULMC †			
H3	PBC → Intention		Supported			
H4	Unnaturalness → Attitude		Inconclusive (underpowered) ‡			
H5	Disgust → Attitude		Supported (marginal under ULMC)			
H6	Ethics → Attitude		Supported			
RQ1	Health Concern → Attitude		Not supported—true null §			

Note. Bayes mean = posterior mean from PyMC (4 chains × 2000 tune + 2000 draws; prior b ~ Normal(0, 1) on standardised coefficients); 95% CrI = equal-tailed 95% credible interval. BF10 = Savage–Dickey Bayes factor against a point-null at β = 0; *BF*_01_ = 1/*BF*_10_ reported in parentheses where evidence favours the null. PLS β = standardised structural path coefficient from PLS-SEM (Wold–Tenenhaus, Mode A, path-weighting inner scheme). Bootstrap 95% CI = percentile interval from 1000 non-parametric bootstrap resamples of the latent SEM, after removal of replicates with |β| > 1.5. Note. † H2 fails Holm correction (*p* = 0.064) and loses significance under ULMC (*p* = 0.111); the path is sensitive to both. ‡ H4 was not statistically supported but the observed effect (β = 0.133) lay below the minimum detectable effect at conventional power (MDE = 0.227 at α = 0.05, power = 0.80) and the Bayes factor evidence for the null was moderate (*BF*_01_ = 3.3); see Table 6 for the corresponding power analysis. § RQ1 was robustly non-significant and the Bayesian evidence in favour of the null was strong (*BF*_01_ = 13).

**Table 6:** Sensitivity power analysis and general dominance decomposition of explained variance.

Panel A. Post hoc sensitivity power for each structural path (*N* = 255).							
Hypothesis	Path	β	*SE*	Power α = 0.05	Power α = 0.05/6	MDE α = 0.05	MDE α = 0.05/6
H1	Attitude → Intention	0.295	0.084	0.94	0.81	0.236	0.293
H2	Subjective Norms → Intention	0.263	0.119	0.60	0.34	0.332	0.413
H3	PBC → Intention	0.609	0.115	1.00	1.00	0.322	0.400
H4	Unnaturalness → Attitude	0.133	0.081	0.38	0.16	0.227	0.282
H5	Disgust → Attitude	−0.272	0.081	0.92	0.77	0.226	0.281
H6	Ethics → Attitude	0.487	0.056	1.00	1.00	0.157	0.195
RQ1	Health Concern → Attitude	0.025	0.076	0.06	0.01	0.213	0.265
Panel B. Budescu general dominance decomposition.							
Outcome (Full *R*^2^)		Predictor		General Dominance		% of Full *R*^2^	
Intention (full *R*^2^ = 0.699)		Subjective Norms		0.2711		38.8%	
Intention (full *R*^2^ = 0.699)		PBC		0.2293		32.8%	
Intention (full *R*^2^ = 0.699)		Attitude		0.1988		28.4%	
Attitude (full *R*^2^ = 0.286)		Ethics		0.2266		79.3%	
Attitude (full *R*^2^ = 0.286)		Disgust		0.0377		13.2%	
Attitude (full *R*^2^ = 0.286)		Health Concern		0.0124		4.3%	
Attitude (full *R*^2^ = 0.286)		Unnaturalness		0.0089		3.1%	

Note. β = standardised path coefficient. SE = realistic standard error from the bootstrap distribution (Table 5, Panel A). Power = post hoc Wald-type two-sided power at the listed α; α = 0.05/6 corresponds to the most conservative step of the Holm–Bonferroni correction within the confirmatory family H1–H6. MDE = minimum detectable standardised coefficient at power = 0.80 and the listed α. Paths with power < 0.80 are interpreted as potentially underpowered; the most consequential case is H4 (Unnaturalness → Attitude), for which the observed β (0.133) lies below the MDE at α = 0.05 (0.227), indicating that the non-significant finding may reflect inadequate power rather than a true null. Note. General dominance values are computed as the average incremental *R*^2^ across all 2^p−1^ subsets of co-predictors [41]. The values sum (subject to rounding) to the full-model *R*^2^ for each outcome. The ranking that emerges from the dominance decomposition for Intention (Subjective Norms > PBC > Attitude) differs from the ranking implied by the standardised SEM path coefficients (PBC > Attitude > Subjective Norms), reflecting the high shared variance among the three TPB predictors. For the Attitude model the dominance and SEM rankings are concordant (Ethics dominates, followed by Disgust).

## Data Availability

The original contributions presented in this study are included in the article/Supplementary Materials. The data presented in this study are openly available in Zenodo at https://doi.org/10.5281/zenodo.20626634 (access date: 29 May 2026), reference number [58]. Further inquiries can be directed to the corresponding author.

## Supplementary Materials

The following supporting information can be downloaded at: https://www.mdpi.com/article/10.3390/foods15142474/s1, File S1: Survey questionnaire; Table S1: Item-level psychometric diagnostics for multi-item scales (*N* = 255); Table S2: Bootstrap distribution of structural path coefficients (1000 resamples); Table S3: Sensitivity power analysis—full results; Table S4a: Logistic regression on dichotomised Intention (Intention > 4); Table S4b: Logistic regression on dichotomised Attitude (Attitude > 4); Table S5a: Elastic-net regression coefficients (10-fold cross-validation); Table S5b: Random-forest permutation importance; Table S6a: PLS-SEM outer loadings; Table S6b: PLS-SEM inner-model summary; Table S7: Bayesian path model—MCMC convergence diagnostics; Table S8a: MANOVA on the joint vector of Attitude and Intention across dietary patterns; Table S8b: Univariate ANOVA and Kruskal-Wallis for Attitude and Intention across dietary patterns; Table S9: Sensitivity of the structural model to the operationalisation of Perceived Behavioural Control (single-indicator analysis); Table S10: Comparison of the parallel baseline model with the alternative hierarchical model (Subjective Norms → Perceived Behavioural Control → Intention).

## References

[1] Guan X., Lei Q., Yan Q., Li X., Zhou J., Du G., Chen J. (2021). Trends and ideas in technology, regulation and public acceptance of cultured meat. Future Foods.

[2] Bryant C.J., Barnett J. (2020). Consumer acceptance of cultured meat: An updated review (2018–2020). Appl. Sci..

[3] Verbeke W., Sans P., Van Loo E.J. (2015). Challenges and prospects for consumer acceptance of cultured meat. J. Integr. Agric..

[4] Szendrő K. (2025). Consumer perceptions of lab-grown cells: Awareness, barriers, and the power of information. A review. Czech J. Anim. Sci..

[5] Raverta P., Sandi I., Martin B., Loera B. (2025). Unfamiliar familiarity: A scoping review on the role of familiarity in consumer acceptance of cultivated meat. Appetite.

[6] Hopkins P.D. (2015). Cultured meat in Western media: The disproportionate coverage of vegetarian reactions, demographic realities, and implications for cultured meat marketing. J. Integr. Agric..

[7] Ajzen I. (1991). The Theory of Planned Behavior. Organ. Behav. Hum. Decis. Process..

[8] Siegrist M., Hartmann C. (2020). Perceived naturalness, disgust, trust and food neophobia as predictors of cultured meat acceptance in ten countries. Appetite.

[9] Wilks M., Phillips C.J.C., Fielding K., Hornsey M.J. (2019). Testing potential psychological predictors of attitudes towards cultured meat. Appetite.

[10] Wilks M., Crimston C.R., Hornsey M.J. (2024). Meat and morality: The moral foundation of purity, but not harm, predicts attitudes toward cultured meat. Appetite.

[11] Dupont J., Harms T., Fiebelkorn F. (2022). Acceptance of cultured meat in Germany—Application of an extended Theory of Planned Behaviour. Foods.

[12] Weinrich R., Strack M., Neugebauer F. (2020). Consumer acceptance of cultured meat in Germany. Meat Sci..

[13] Li H., Van Loo E.J., Van Trijp H.C.M., Chen J., Bai J. (2023). Will cultured meat be served on Chinese tables? A study of consumer attitudes and intentions about cultured meat in China. Meat Sci..

[14] Huang S., Uehara T. (2023). Young consumers’ perceptions of and preferences for alternative meats: An empirical study in Japan and China. Front. Sustain. Food Syst..

[15] Hibino A., Nakamura F., Furuhashi M., Takeuchi S. (2023). How can the unnaturalness of cellular agricultural products be familiarized?: Modeling public attitudes toward cultured meats in Japan. Front. Sustain. Food Syst..

[16] Lazou A.E., Revelou P.K., Kougioumtzoglou S., Strati I.F., Kanellou A., Batrinou A. (2024). Cultured meat: A survey of awareness among Greek consumers. AIMS Agric. Food.

[17] Chriki S., Alhujaili A., Hallman W.K., Payet V., Ellies-Oury M., Hocquette J. (2024). Attitudes toward artificial meat in Arab countries. J. Food Sci..

[18] Rodríguez Escobar M.I., Han S., Cadena E., De Smet S., Hung Y. (2025). Cross-cultural consumer acceptance of cultured meat: A comparative study in Belgium, Chile, and China. Food Qual. Prefer..

[19] Engel L., Vilhelmsen K., Richter I., Moritz J., Ryynänen T., Young J.F., Burton R.J.F., Kidmose U., Klöckner C.A. (2024). Psychological factors influencing consumer intentions to consume cultured meat, fish and dairy. Appetite.

[20] Maqsood S., Ajayi F.F., Mostafa H., Lawal K.G., Mubaiwa J., Alantali N., Alshihhi M., Aldhaheri M. (2025). Are Emirati consumers in United Arab Emirates open to alternative proteins? Insights into their attitudes and willingness to replace animal protein sources. Front. Sustain. Food Syst..

[21] Bryant C., Szejda K., Parekh N., Deshpande V., Tse B. (2019). A survey of consumer perceptions of plant-based and clean meat in the USA, India, and China. Front. Sustain. Food Syst..

[22] Choudhary F., Khandi S.A., Hassoun A., Aadil R.M., Bekhit A.E.D.A., Abdi G., Bhat Z.F. (2024). Awareness and acceptance of informed and professional consumers of Jammu and Kashmir about cultured meat. Appl. Food Res..

[23] Šostar M., Joy J., Ramanathan H.N. (2025). Consumer trust in emerging food technologies: A comparative analysis of Croatia and India. Sustainability.

[24] Lewisch L., Riefler P. (2023). How social norms and dietary identity affect willingness to try cultured meat. Br. Food J..

[25] World Medical Association (2013). World Medical Association Declaration of Helsinki: Ethical principles for medical research involving human subjects. JAMA.

[26] Kline R.B. (2016). Principles and Practice of Structural Equation Modeling.

[27] Cohen J. (1988). Statistical Power Analysis for the Behavioral Sciences.

[28] Bryant C., Dillard C. (2019). The impact of framing on acceptance of cultured meat. Front. Nutr..

[29] Ajzen I. (2006). Constructing a Theory of Planned Behavior Questionnaire. https://people.umass.edu/~aizen/pdf/tpb.measurement.pdf.

[30] Ajzen I. (2002). Perceived behavioral control, self-efficacy, locus of control, and the Theory of Planned Behavior. J. Appl. Soc. Psychol..

[31] McDonald R.P. (1999). Test Theory: A Unified Treatment.

[32] Hair J.F., Black W.C., Babin B.J., Anderson R.E. (2019). Multivariate Data Analysis.

[33] Bentler P.M., Bonett D.G. (1980). Significance tests and goodness of fit in the analysis of covariance structures. Psychol. Bull..

[34] Hu L., Bentler P.M. (1999). Cutoff criteria for fit indexes in covariance structure analysis: Conventional criteria versus new alternatives. Struct. Equ. Model..

[35] Podsakoff P.M., MacKenzie S.B., Lee J.-Y., Podsakoff N.P. (2003). Common method biases in behavioral research: A critical review of the literature and recommended remedies. J. Appl. Psychol..

[36] Williams L.J., Hartman N., Cavazotte F. (2010). Method variance and marker variables: A review and comprehensive CFA marker technique. Organ. Res. Methods.

[37] Hayes A.F. (2018). Introduction to Mediation, Moderation, and Conditional Process Analysis: A Regression-Based Approach.

[38] Preacher K.J., Hayes A.F. (2008). Asymptotic and resampling strategies for assessing and comparing indirect effects in multiple mediator models. Behav. Res. Methods.

[39] Tabachnick B.G., Fidell L.S. (2013). Using Multivariate Statistics.

[40] Azen R., Budescu D.V. (2003). The dominance analysis approach for comparing predictors in multiple regression. Psychol. Methods.

[41] Budescu D.V. (1993). Dominance analysis: A new approach to the problem of relative importance of predictors in multiple regression. Psychol. Bull..

[42] Malavalli M.M., Hamid N., Kantono K., Liu Y., Seyfoddin A. (2021). Consumers’ Perception of In-Vitro Meat in New Zealand Using the Theory of Planned Behaviour Model. Sustainability.

[43] Lanz M., Wassmann B., Siegrist M. (2025). Cultured meat: Vegetarian or not? Exploring young vegetarians’ and omnivores’ perceptions of this new technology. Appetite.

[44] Herziger A. (2024). Moving beyond meat: Perceived unnaturalness and disgust across cultured foods. J. Environ. Psychol..

[45] Loera B., Raverta P., Bertero A., Cresti M., Stano S., Lo Sapio L. (2026). Beyond borders: A cross-national study on cultivated meat acceptance in Italy, France, and the Netherlands. Innov. Food Sci. Emerg. Technol..

[46] Wilks M., Hornsey M., Bloom P. (2021). What does it mean to say that cultured meat is unnatural?. Appetite.

[47] Kass R.E., Raftery A.E. (1995). Bayes factors. J. Am. Stat. Assoc..

[48] Lee M.D., Wagenmakers E.-J. (2013). Bayesian Cognitive Modeling: A Practical Course.

[49] Sathyamala C. (2019). Meat-eating in India: Whose food, whose politics, and whose rights?. Policy Futures Educ..

[50] Prakash J., Krishnamurti R.Y. (2020). The dietary practices and food-related rituals in Indian tradition and their role in health and nutrition. Nutritional and Health Aspects of Food in South Asian Countries.

[51] Ahsan M., Uzair M., Ali M. (2021). Attitudes and perceptions towards cultured meat among general population in Pakistan. ASEAN J. Sci. Eng. Educ..

[52] Rosenfeld D.L., Tomiyama A.J. (2022). Would you eat a burger made in a petri dish? Why people feel disgusted by cultured meat. J. Environ. Psychol..

[53] Arango L., Septianto F., Pontes N. (2024). The role of conventional meat unnaturalness in cultured meat acceptance: A test of holistic mindset. Appetite.

[54] Hamdan M.N., Ramli M.A., Zaman Huri N.M.F., Abd Rahman N.N.H., Abdullah A. (2021). Will Muslim consumers replace livestock slaughter with cultured meat in the market?. Trends Food Sci. Technol..

[55] Tenenhaus M., Vinzi V.E., Chatelin Y.-M., Lauro C. (2005). PLS path modeling. Comput. Stat. Data Anal..

[56] Wold H., Jöreskog K.G., Wold H. (1982). Soft modeling: The basic design and some extensions. Systems Under Indirect Observation: Causality, Structure, Prediction, Part II.

[57] Sheeran P. (2002). Intention–behavior relations: A conceptual and empirical review. Eur. Rev. Soc. Psychol..

[58] Farooqui N., Kaczmarek A.M. (2026). Perceived behavioural control and animal-welfare ethics predict cultured meat acceptance in India: An extended Theory of Planned Behaviour analysis. Preprints.

