# Supplementary material for: Perceived Behavioural Control and Animal-Welfare Ethics Predict Cultured-Meat Acceptance in INDIA: An Extended Theory of Planned Behaviour Analysis
Source: Foods. 2026 Jul 13;15(14):2474. doi: 10.3390/foods15142474 (PMC13408475; doi:10.3390/foods15142474)
Supplement: Supplementary file 1 [file foods-15-02474-s001.zip › foods-4377079-supplementary.pdf]

Supplementary Materials

Perceived behavioural control and animal-welfare ethics drive cultured meat acceptance in India: An extended Theory of Planned Behaviour analysis

Nematullah Farooqui, Anna Kaczmarek

These Supplementary Materials contain the full survey questionnaire and ten supplementary tables that complement the main-text results. A reproducibility package — Python scripts, pinned requirements file, and cleaned analytic dataset — is provided separately as a code/data archive.

File S1. Survey questionnaire

The instrument was administered in English via Google Forms. The questionnaire consisted of eight sections (A–H). Items in Sections C–G were rated on 7-point scales as indicated below.

Section A. Prior awareness of cultured meat

Q1. Before today, had you heard about cultured meat — meat produced from animal cells without slaughter? (Yes / No)

Section B. Information note

Respondents were presented with the following neutrally worded definition before the substantive items:

"Cultured meat is real animal meat produced by cultivating animal cells in a controlled environment (a bioreactor), without raising or slaughtering live animals. The final product is intended to resemble conventional meat in taste, texture, and nutritional content. Cultured meat is currently in early commercial development; regulatory approvals have been granted in a small number of countries."

Section C. Attitude toward trying cultured meat (ATT)

Semantic differential, 7-point bipolar scales. Stem: "Trying cultured meat would be…"

A1. negative — positive

A2. unpleasant — pleasant

A3. unattractive — attractive

A4. not worthwhile — worthwhile

Section D. Subjective Norms (SN)

7-point Likert (1 = strongly disagree, 7 = strongly agree).

D1. "People who are important to me would support me trying cultured meat."

D2. "People whose opinions I value would approve of me trying cultured meat."

Section E. Perceived Behavioural Control (PBC)

7-point Likert (1 = strongly disagree, 7 = strongly agree).

E1. "If I wanted to, I could easily try cultured meat once it becomes available."

E2. "Whether I try cultured meat is entirely up to me."

Section F. Behavioural intention (INT)

7-point Likert (1 = strongly disagree, 7 = strongly agree).

F1. “I intend to try cultured meat as soon as I have the opportunity.”

F2. “I would buy cultured meat at least once if it were available at a reasonable price.”

Section G. Psychological antecedents of attitude

7-point Likert (1 = strongly disagree, 7 = strongly agree).

G1 (Perceived Unnaturalness). “Cultured meat seems unnatural to me.”

G2 (Disgust). “The idea of eating cultured meat feels disgusting.”

G3 (Ethics — animal welfare). “Cultured meat could reduce animal suffering.”

G4 (Health Concern). “I am concerned about unknown long-term effects of cultured meat on human health.”

Section H. Demographics and background

H1. Age (open numeric).

H2. Gender (Female / Male / Other / Prefer not to say).

H3. Dietary pattern (I eat meat regularly / I limit meat consumption / Vegetarian / Vegan / Other).

H4. Country of residence (open text).

H5. Education level (Primary or lower secondary / Vocational or technical / High school / Bachelor's degree / Master's degree or higher).

H6. Place of residence (Rural area / Town < 20,000 / City 20,000–100,000 / City 100,000–500,000 / City > 500,000).

H7. Self-rated financial situation (1 = very bad to 7 = very good).

Supplementary Tables

Table S1. Item-level psychometric diagnostics for multi-item scales (*N* = 255).

Scale	Item	n	Mean	*SD*	Item-total r (corrected)	Alpha (full scale)	Alpha if item deleted
Attitude (ATT)	A1	255	3.3961	2.0515	0.8824	0.9495	0.9326
Attitude (ATT)	A2	255	3.3255	1.9237	0.8948	0.9495	0.9287
Attitude (ATT)	A3	255	3.3765	1.9541	0.8814	0.9495	0.9326
Attitude (ATT)	A4	255	3.3333	1.9709	0.8533	0.9495	0.9411
Subjective Norms (SN)	D1	255	3.4078	2.0674	0.8761	0.9340	n.a.
Subjective Norms (SN)	D2	255	3.5333	2.0900	0.8761	0.9340	n.a.
Perceived Behavioural Control (PBC)	E1	255	3.7804	2.2323	0.5363	0.6979	n.a.
Perceived Behavioural Control (PBC)	E2	255	4.6275	2.1678	0.5363	0.6979	n.a.
Intention (INT)	F1	255	3.3608	2.1359	0.8310	0.9070	n.a.
Intention (INT)	F2	255	3.7843	2.2511	0.8310	0.9070	n.a.

Note. Single-item measures (G1–G4) do not permit reliability estimation and are not included. α = Cronbach's alpha; corrected *r* = item-total correlation with the remaining items of the same scale; α if item deleted is undefined for two-item scales because removing one item leaves a single indicator.

Table S2. Bootstrap distribution of structural path coefficients (1,000 resamples).

Hypothesis	Path	Std. estimate (original)	Bootstrap mean	Bootstrap median	Bootstrap bias	Bootstrap *SE*	95% CI lo (percentile)	95% CI hi (percentile)	95% CI lo (bias-corrected)	95% CI hi (bias-corrected)	Bootstrap p (two-sided)	Replicates used	Extreme replicates removed	Holm-corrected p (boot)	Decision (boot, Holm)
H1	INT ~ ATT	0.2955	0.2987	0.2973	0.0033	0.0842	0.1421	0.4633	0.1356	0.4568	0.0000	1000	0	0.0000	Supported
H2	INT ~ SN	0.2633	0.3010	0.3009	0.0377	0.1186	0.0558	0.5409	-0.0197	0.4655	0.0135	741	259	0.0270	Supported
H3	INT ~ PBC	0.6088	0.5606	0.5615	-0.0482	0.1151	0.3438	0.7782	0.4402	0.8747	0.0000	741	259	0.0000	Supported
H4	ATT ~ G1	0.1331	0.1318	0.1309	-0.0012	0.0811	-0.0291	0.2920	-0.0266	0.2945	0.1060	1000	0	0.1060	Not supported
H5	ATT ~ G2	-0.2715	-0.2713	-0.2707	0.0002	0.0807	-0.4305	-0.1102	-0.4309	-0.1107	0.0020	1000	0	0.0060	Supported
H6	ATT ~ G3	0.4867	0.4888	0.4901	0.0021	0.0560	0.3738	0.5926	0.3695	0.5883	0.0000	1000	0	0.0000	Supported
RQ1	ATT ~ G4	0.0255	0.0231	0.0226	-0.0024	0.0761	-0.1308	0.1704	-0.1261	0.1752	0.7740	1000	0	n.a.	n.a.

Note. Replicates with $|\beta| > 1.5$ were treated as numerical failures and excluded from the summary; "Replicates used" reports the number of stable replicates. Bias = bootstrap mean minus the original point estimate. Holm-corrected p-values apply to the confirmatory family H1–H6.

Table S3. Sensitivity power analysis — full results.

Hypothesis	Path	Observed β	Realistic *SE* (bootstrap)	Power at α = .05	Power at α = .05 / 6 (Holm)	MDE at power = .80, α = .05	MDE at power = .80, α = .05/6	|β| / *SE* ratio	Adequate power at α=.05?	Adequate power at α=.05/6?
H1	INT ~ ATT	0.2955	0.0842	0.9395	0.8084	0.2358	0.2929	3.5103	True	True
H2	INT ~ SN	0.2633	0.1186	0.6025	0.3378	0.3323	0.4127	2.2199	False	False
H3	INT ~ PBC	0.6088	0.1151	0.9996	0.9960	0.3223	0.4004	5.2918	True	True
H4	ATT ~ G1	0.1331	0.0811	0.3751	0.1594	0.2271	0.2821	1.6413	False	False
H5	ATT ~ G2	-0.2715	0.0807	0.9198	0.7660	0.2261	0.2809	3.3640	True	False
H6	ATT ~ G3	0.4867	0.0560	1.0000	1.0000	0.1570	0.1950	8.6859	True	True
RQ1	ATT ~ G4	0.0255	0.0761	0.0629	0.0121	0.2131	0.2647	0.3348	False	False

Note. Power computed under the Wald two-sided test with bootstrap-derived realistic *SE*. MDE = minimum detectable standardised effect at power = .80 under the listed alpha. α = .05/6 corresponds to the most conservative Holm step within the confirmatory family H1–H6.

Table S4a. Logistic regression on dichotomised Intention (Intention > 4).

Predictor	Coef (logit)	*SE* (HC3)	z	p	Odds ratio	OR 95% CI low	OR 95% CI high	Model
const	-1.0909	0.2093	-5.2121	0.0000	0.3359	0.2229	0.5063	Intention > 4 (binary)
Attitude_mean	0.9112	0.2486	3.6653	0.0002	2.4872	1.5279	4.0487	Intention > 4 (binary)
SubjectiveNorms_mean	0.7913	0.2815	2.8108	0.0049	2.2062	1.2706	3.8307	Intention > 4 (binary)
PBC_mean	1.5272	0.3280	4.6560	0.0000	4.6052	2.4213	8.7589	Intention > 4 (binary)

Note. Standard errors are heteroskedasticity-consistent (HC3). Coefficients reported on the logit scale; odds ratios and 95% confidence intervals reported separately. Predictors were standardised before estimation, so the odds ratios refer to a one-*SD* increase in each predictor.

Table S4b. Logistic regression on dichotomised Attitude (Attitude > 4).

Predictor	Coef (logit)	*SE* (HC3)	z	p	Odds ratio	OR 95% CI low	OR 95% CI high	Model
const	-1.1239	0.1762	-6.3788	0.0000	0.3250	0.2301	0.4591	Attitude > 4 (binary)
Unnaturalness	0.1904	0.2248	0.8471	0.3969	1.2097	0.7787	1.8793	Attitude > 4 (binary)
Disgust	-0.5302	0.2152	-2.4639	0.0137	0.5885	0.3859	0.8972	Attitude > 4 (binary)
Ethics	1.1611	0.2250	5.1608	0.0000	3.1936	2.0548	4.9636	Attitude > 4 (binary)
HealthConcern	0.0654	0.1996	0.3276	0.7432	1.0676	0.7219	1.5788	Attitude > 4 (binary)

Note. As above, fitted on the four perceptual predictors (Unnaturalness, Disgust, Ethics, Health Concern). McFadden's pseudo-R^2 and *AIC*/*BIC* are reported in the analysis log.

Table S5a. Elastic-net regression coefficients (10-fold cross-validation).

Predictor	Std. coefficient (ElasticNet)	Retained (|coef| > 0)	Outcome
Attitude_mean	0.2326	True	Intention
SubjectiveNorms_mean	0.3635	True	Intention
PBC_mean	0.2942	True	Intention
Unnaturalness	0.0000	False	Intention
Disgust	0.0000	False	Intention
Ethics	0.0000	False	Intention
HealthConcern	0.0000	False	Intention
H1	-0.0009	True	Intention
Education_code	0.0000	False	Intention
H7	0.0000	False	Intention
Unnaturalness	0.0000	False	Attitude
Disgust	-0.1119	True	Attitude
Ethics	0.4504	True	Attitude
HealthConcern	0.0000	False	Attitude
H1	0.0000	False	Attitude
Education_code	0.0000	False	Attitude
H7	0.0000	False	Attitude

Note. Predictors and outcomes were standardised before estimation. Tuning was performed by 10-fold cross-validation over a grid of l1-ratio values (.1, .3, .5, .7, .9, 1.0) and 50 alpha values per ratio. A coefficient of exactly zero indicates that the elastic net excluded that predictor at the selected tuning parameters.

Table S5b. Random-forest permutation importance.

Predictor	Permutation importance (mean ΔR^2)	Permutation importance (*SD*)	Outcome
SubjectiveNorms_mean	0.3410	0.0242	Intention
PBC_mean	0.2717	0.0238	Intention
Attitude_mean	0.2174	0.0209	Intention
Ethics	0.0700	0.0053	Intention
Disgust	0.0512	0.0035	Intention
Unnaturalness	0.0414	0.0036	Intention
H1	0.0341	0.0022	Intention
H7	0.0276	0.0030	Intention
HealthConcern	0.0265	0.0029	Intention
Education_code	0.0142	0.0012	Intention

Ethics	0.6572	0.0567	Attitude
Disgust	0.3533	0.0256	Attitude
Unnaturalness	0.2630	0.0233	Attitude
H1	0.2368	0.0233	Attitude
HealthConcern	0.2045	0.0223	Attitude
H7	0.1523	0.0142	Attitude
Education_code	0.0936	0.0112	Attitude

Note. Permutation importance computed over 50 permutations per predictor. The reported value is the mean decrease in out-of-sample R^2 when the predictor's column is shuffled.

Table S6a. PLS-SEM outer loadings.

Latent variable	Manifest variable	Outer loading
Unnat	G1	1.0000
Disg	G2	1.0000
Eth	G3	1.0000
HC	G4	1.0000
SN	D1	0.9675
SN	D2	0.9696
PBC	E1	0.9318
PBC	E2	0.8060
ATT	A1	0.9361
ATT	A2	0.9427
ATT	A3	0.9347
ATT	A4	0.9150
INT	F1	0.9584
INT	F2	0.9552

Note. Outer loadings = correlation between each indicator and its latent construct in the PLS-SEM model. Single-indicator latents (Unnat, Disg, Eth, HC) have outer loadings fixed at 1.0 by construction.

Table S6b. PLS-SEM inner-model summary.

Latent variable	R^2	AVE	Composite reliability
ATT	0.2855	0.8690	0.9637
Disg	n.a.	1.0000	1.0000
Eth	n.a.	1.0000	1.0000
HC	n.a.	1.0000	1.0000
INT	0.7119	0.9155	0.9559
PBC	n.a.	0.7590	0.8624
SN	n.a.	0.9381	0.9680
Unnat	n.a.	1.0000	1.0000

Note. R^2 = proportion of variance in the endogenous latent variable explained by its predictors; reported only for endogenous LVs (ATT, INT). AVE = average variance extracted; CR = composite reliability (ρ_c).

Table S7. Bayesian path model — MCMC convergence diagnostics.

equation	parameter	mean	sd	eti89_lb	eti89_ub	ess_bulk	ess_tail	r_hat	mcse_mean	mcse_sd
Intention	b_ATT	0.2530	0.0491	0.1700	0.3300	6107	5428	1.0000	0.0006	0.0005
Intention	b_SN	0.3780	0.0570	0.2800	0.4700	5372	4520	1.0000	0.0008	0.0006
Intention	b_PBC	0.3200	0.0483	0.2400	0.4000	6330	4874	1.0000	0.0006	0.0004
Attitude	b_U	0.1300	0.0810	0.0001	0.2600	5651	5296	1.0000	0.0011	0.0008
Attitude	b_D	-0.2630	0.0770	-0.3900	-0.1400	5350	5762	1.0000	0.0011	0.0007
Attitude	b_E	0.4740	0.0590	0.3800	0.5700	6163	5753	1.0000	0.0008	0.0005
Attitude	b_H	0.0230	0.0730	-0.0940	0.1400	5644	5531	1.0000	0.0010	0.0007

Note. Diagnostics computed in arviz. R-hat ≤ 1.01 and ESS ≥ 400 indicate adequate convergence; all chains satisfied both criteria. Equation 1 = Intention regressed on ATT/SN/PBC; Equation 2 = Attitude regressed on the four perceptual predictors.

Table S8a. MANOVA on the joint vector of Attitude and Intention across dietary patterns.

Test	Value	Num DF	Den DF	F Value	Pr > F	Significant
Wilks' lambda	0.9882	4	494.00	0.7342	0.5689	No
Pillai's trace	0.0118	4	496.00	0.7368	0.5672	No
Hotelling-Lawley trace	0.0119	4	295.36	0.7336	0.5697	No
Roy's greatest root	0.0085	2	248.00	1.0504	0.3514	No

Note. Three dietary groups included in the parametric MANOVA: Omnivore (n = 161), Flexitarian (n = 73), Vegetarian (n = 17). Vegans (n = 4) excluded from parametric tests due to insufficient sample size.

Table S8b. Univariate ANOVA and Kruskal-Wallis for Attitude and Intention across dietary patterns.

Outcome	Test	Statistic	*df*	p	η^2	ω^2	Cohen's f	ε^2
Attitude	ANOVA	$F = 0.43$	2, 248	0.6540	0.0030	0.0000	0.0590	—
Attitude	Kruskal-Wallis	H = 7.33	3	0.0620	—	—	—	0.0170
Intention	ANOVA	$F = 0.78$	2, 248	0.4610	0.0060	0.0000	0.0790	—
Intention	Kruskal-Wallis	H = 4.13	3	0.2480	—	—	—	0.0050

Note. ANOVA excludes Vegans (n = 4); Kruskal-Wallis includes all four groups. All effect sizes are negligible ($\eta^2 < .01$). ε^2 = epsilon-squared (non-parametric effect size). Hypothesis H7 was therefore not supported under either parametric or non-parametric tests.

Table S9. Sensitivity of the structural model to the operationalisation of Perceived Behavioural Control (single-indicator analysis).

Model	H1 (ATT→INT) β	H1 (ATT→INT) p	H2 (SN→INT) β	H2 (SN→INT) p	H3 (PBC→INT) β	H3 (PBC→INT) p	*CFI*	*TLI*	*RMSEA*	chi2	DoF	*AIC*	*BIC*
A: PBC = E1 + E2 (two-indicator latent, reference)	0.295	0.000	0.263	0.032	0.609	0.000	0.920	0.901	0.110	313.50	77	53.54	152.70
B: PBC = E1 only (single indicator)	0.331	0.000	0.432	0.000	0.552	0.000	0.886	0.861	0.137	390.31	68	42.94	124.39
C: PBC = E2 only (single indicator)	0.388	0.000	0.675	0.000	0.138	0.003	0.923	0.906	0.109	274.41	68	43.85	125.30

Note. Each row re-estimates the latent SEM with PBC represented differently: Model A uses both indicators (E1, E2); Models B and C use a single indicator (E1 or E2 alone) treated as a perfect measure of the construct. β = standardised path coefficient; *p* = two-sided Wald p-value. The PBC → Intention path remained positive and significant in every specification, confirming that the core structural conclusions do not depend on the two-item operationalisation. *CFI*, *TLI*, *RMSEA* = global fit indices; *AIC*, *BIC* = information criteria.

Table S10. Comparison of the parallel baseline model with the alternative hierarchical model (Subjective Norms → Perceived Behavioural Control → Intention).

Model	ATT→INT β	ATT→INT p	SN→INT β	SN→INT p	PBC→INT β	PBC→INT p	chi2	DoF	*CFI*	*TLI*	*RMSEA*	*AIC*	*BIC*	SN→PBC β	SN→PBC p
M1: parallel (INT ~ ATT + SN + PBC)	0.295	0.000	0.263	0.032	0.609	0.000	313.50	77	0.920	0.901	0.110	53.54	152.70	n.a.	n.a.
M2: hierarchical (SN → PBC → INT)	0.313	0.000	n.a.	n.a.	0.859	0.000	316.33	78	0.920	0.902	0.110	51.52	147.13	0.865	0.000

Note. M1 = parallel baseline (Intention regressed on Attitude, Subjective Norms, and PBC). M2 = hierarchical model (Subjective Norms specified as an antecedent of PBC). β = standardised path coefficient; *p* = two-sided Wald p-value; cells marked n.a. denote a path not present in that model. The two models showed equivalent global fit, and the lower *AIC* and *BIC* of M2 indicate a marginal preference for the hierarchical specification.
